# Real-Time Shear Wave versus Transient Elastography for Predicting Fibrosis: Applicability, and Impact of Inflammation and Steatosis. A Non-Invasive Comparison

**DOI:** 10.1371/journal.pone.0163276

**Published:** 2016-10-05

**Authors:** Thierry Poynard, Tam Pham, Hugo Perazzo, Mona Munteanu, Elena Luckina, Djamel Elaribi, Yen Ngo, Luminita Bonyhay, Noemie Seurat, Muriel Legroux, An Ngo, Olivier Deckmyn, Dominique Thabut, Vlad Ratziu, Olivier Lucidarme, FIBROFRANCE-HECAM

**Affiliations:** 1 Hepato-Gastroenterology, APHP UPMC Liver Center, Paris, France; 2 INSERM, UMR S938, Paris, France; 3 Hepatology Research Unit, BioPredictive, Paris, France; 4 Radiology, APHP UPMC Liver Center, Paris, France; Yonsei University College of Medicine, REPUBLIC OF KOREA

## Abstract

**Background and Aims:**

Real-time shear wave elastography (2D-SWE) is a two-dimensional transient elastography and a competitor as a biomarker of liver fibrosis in comparison with the standard reference transient elastography by M probe (TE-M). The aims were to compare several criteria of applicability, and to assess inflammation and steatosis impact on elasticity values, two unmet needs.

**Methods:**

We took FibroTest as the fibrosis reference and ActiTest and SteatoTest as quantitative estimates of inflammation and steatosis. After standardization of estimates, analyses used curve fitting, quantitative Lin concordance coefficient [LCC], and multivariate logistic regression.

**Results:**

A total of 2,251 consecutive patients were included. We validated the predetermined 0.2 kPa cut-off as a too low minimal elasticity value identifying not-reliable 2D-SWE results (LCC with FibroTest = 0.0281[-0.119;0.175]. Other criteria, elasticity CV, body mass index and depth of measures were not sufficiently discriminant. The applicability of 2D-SWE (95%CI) 89.6%(88.2–90.8), was significantly higher than that of TE, 85.6%(84.0–87.0; P<0.0001). In patients with non-advanced fibrosis (METAVIR F0F1F2), elasticity values estimated by 2D-SWE was less impacted by inflammation and steatosis than elasticity value estimated by TE-M: LCC (95%CI) 0.039 (0.021;0.058) vs 0.090 (0.068;0.112;P<0.01) and 0.105 (0.068;0.141) vs 0.192 (0.153;0.230; P<0.01) respectively. The three analyses methods gave similar results.

**Conclusions:**

Elasticity results including very low minimal signal in the region of interest should be considered not reliable. 2D-SWE had a higher applicability than TE, the reference elastography, with less impact of inflammation and steatosis especially in patients with non-advanced fibrosis, as presumed by blood tests.

**Trial Registration:**

ClinicalTrials.gov NCT01927133

## Introduction

Liver fibrosis evaluation using real-time shear wave elastography (2D-SWE) by Aixplorer^TM^ is a two-dimensional transient elastography technique, [1] which is a competitor of the transient elastography with probe M (TE-M) considered as a standard. [2]

2D-SWE estimates the speed of a shear wave to provide a quantitative estimate of tissue stiffness. 2D-SWE has the advantage over TE of being able to image liver stiffness in real time, not limited at a single location, and guided by a higher frame-rate B-mode image.[1,3] Two disadvantages were also identified and related to applicability and reliability: "quality criteria not well defined" and the "influence of inflammation" should be clarified.[2]

The first aim was to better define quality criteria [2,3,4,5,6] (**S1 Table**) The secondary aim was to better quantify the impact of inflammation and steatosis on elasticity values, independently of fibrosis value.

These aims were reachable more rapidly and in larger populations using validated blood tests as reference, rather than using liver biopsies.

## Methods

### Patients

**Fig 1:**
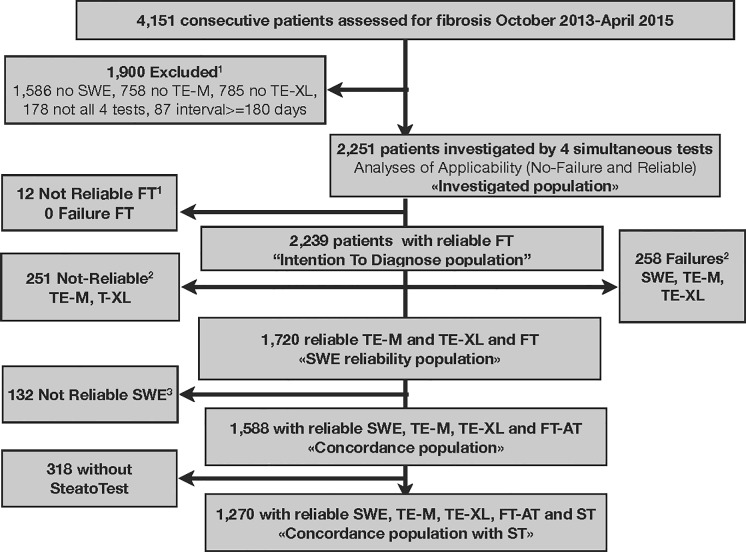
Subjects Flow chart. ^1^ As FibroTest (FT) was taken as the reference, the not-reliable FTs were excluded of the "intention to diagnose population". ^2^ Several failures or not-reliable results were possible in the same patient explaining why the total failures or not reliable results were greater than the number of patients excluded of the 2D-SWE reliability population and of the concordance population. ^3^ 132 patients had not-reliable SWE, but reliable TE-M and TE-XL and FT.

Consecutive patients undergoing chronic liver disease assessment at the "Groupe Hospitalier Pitié Salpêtrière" Hospital in Paris, France were recruited **(Fig 1)**. We included patients aged 18 years or older who had undergone simultaneous serum sampling for FibroTest and attempted liver stiffness measurements with 2D-SWE and TE-M and TE-XL.(**S1 File)**

Patients with acute liver disease, ALT greater than 622 IU/L, and extra-hepatic cholestasis, were not included. Written informed consent have been obtained for all patients and all clinical investigations have been conducted according to the principles expressed in the Declaration of Helsinki. The ethic committee of Groupe Hospitalier Pitié Salpêtrière has approved the research. This study is a sub-project of the FibroFrance project (NCT01927133). All co-authors had access to the study data and had reviewed and approved the final manuscript.

### Elasticity measurements

2D-SWE was performed using the Aixplorer^TM^ ultrasound system (Supersonic Imagine S.A., Aix-en-Provence, France). For each patient the mean, and the median of Qbox elasticity were assessed, as well as the lowest and the highest elasticity values. A single estimate of the Qbox elasticity was performed, as it has been previously validated using biopsy that less than five measures were sufficient for 2D-SWE in comparison with TE, three measures [7,8] and finally one measure [9].

TE-M and TE-XL were performed using M and XL probes respectively, using FibroScan^TM^ (Echosens, Paris, France) according to the instructions and training provided by the manufacturer.[2] Steatosis was also assessed using the controlled attenuation parameter (CAP) of TE-M measures.[10] The measurements were not blinded as the same operator performed successively TE-M, TE-XL and 2D-SWE, but blinded to the blood tests results.

### Blood test measurements (S2 File)

FibroTest, ActiTest and SteatoTest were performed according to the manufacturer’s’ recommendations, using the usual predetermined cutoffs [11,12,13]. FibroTest, ActiTest and SteatoTest (BioPredictive Paris, France; FibroSURE LabCorp Burlington, NC, USA) were algorithms including 5 to 10 components adjusted for age and gender.

### Definition of applicability rate

For FibroTest, a measurement was classified as a failure when serum sampling was impossible; it was classified as not-reliable if one component in the measurement had an extreme value, which induced a change of more than 0.30 in the FibroTest value when calculated using the usual median instead.[11]

For TE-M and TE-XL, signal absence was considered a failure, and the standard reliability definition was the IQR/liver stiffness measurement (IQR/M) <0.30, at least 10 measurements and a success rate of 60% or greater.[2,14]

For 2D-SWE, there were no standard,[2,3,4,5] and we compared the different definitions published,**(S1 and S2 Tables)** using the “strength of concordance” method detailed elsewhere.[4,6]. In the absence of reference, measurement of the strength of the concordance between two imperfect gold standards could be used as a tool for identifying factors of variability. Any variability factor of one test should impact the strength of the association between the two tests, assuming that this variability factor is not also associated with the other test (independent tests).

### Impact of inflammation and steatosis on elasticity

The strength of concordance between 2D-SWE and FibroTest were estimated using the Lin concordance coefficient of correlation (LCC), stratified according to presence of significant steatosis and significant inflammation (**S2 File and S3 Table)**

### Statistical analysis

The aims of this study were not to assess the performance of 2D-SWE versus TE-M, the standard of elastography.

**Fig 2:**
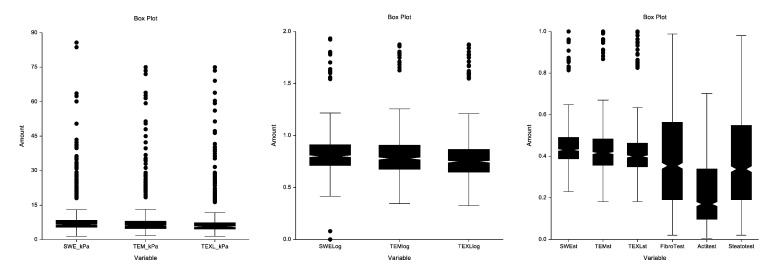
Distribution of elasticity values according to different standardizations. Upper circles corresponded to severe outliers for whom elasticity values were outside boundaries defined as three times the inter-quartile range. (A) No transformation. (B) Log^10^ Transformation. (C) Log-transformation plus standardized according to the range of values (0-75kPa), from 0.00 to 1.00. (n = 1,588 for all tests except for ActiTest n = 1,270).

Firstly, we attempt to normalize and standardize the expression of elasticity. We assessed the impact of elasticity expression in kPa without and with transformation to reduce skewed distribution. For the concordance analyzes we transform the TE-M, TE-XL and 2D-SWE elasticity, first by logarithmic transformation, and secondly by standardizing the expression from 0 to 1, dividing each value by 74, the range between 1 to 75 kPa **(Fig 2)**. We checked that indeed the non-transformation of data would had induced higher coefficient of variation (CV) and lower concordances (S3 **Table**).

Secondly, we describe the association between elasticity values and fibrosis severity using curve fitting. We compared graphically and with squared correlation coefficient (R2)**(S4 Table),** the simplest continuous linear model, with models assuming several parts in correlation. The rational was that elasticity values, despite Log-transformation and standardization, were still increasing in patients with stages F3 and F4 in comparison with patients with non-severe stages (F0F1F2).

Thirdly, we used LCC with bilateral 95% confidence interval, for assessing the quantitative strength of concordance between elasticity estimates, FibroTest measures being taken as reference. [15]

LCC was also used to compare the impact of inflammation and steatosis on elasticity values. A quantitative multivariate regression analysis was performed using the diagnostic of F3F4 as the endpoint. We compared the regression coefficients as well as the area under the ROC curves (AUROC) of the logistic regression function, including elasticity values, inflammation and steatosis and the cause of liver disease as variability factors. Due to the relatively small number of patients, only NAFLD and CHC were entered in these models.

The AUROC were estimated by the non-parametric method, and compared using the paired method of Zhou et al.[16] Recommendations have been made for assessing the intention to diagnose, to use the worse scenario for missing data.[17,18] Accordingly, we used for missing elasticity measure the ([1-standardized reference measure], that is [1-FibroTest]). We used NCSS software (Kaysville, Utah, USA) [19] for standard statistics and LCC.

## Results

### Populations included

Between October 2013 and April 2015, 4,151 consecutive patients were assessed for fibrosis, 2,251 patients constituted the "intention-to-diagnose population", 1,720 patients constituted the "SWE reliability population", 1,558 patients the "concordance population", 1,270 patients the "concordance population with SteatoTest" and 663 patients were the "not-applicable population".

There were no major, or unexpected differences, between the different populations characteristics.**(S5–S9 Tables)**. Only 53/1588 (3.3%) patients had an interval between blood tests and elasticity measurements between one to six months.

### Standardization of elasticity values

The standardization of elasticity values (**Fig 2. Panel A**) by using Log10 transformation (**Fig 2. Panel B**) and limiting the maximum to 75 kPa and dividing by 75, permitted to obtain as for FibroTest a similar range between 0.00 to 1.00 as well as less skewed distribution (**Fig 2. Panel C**).

The lowest CV was observed for "Mean of standardized elasticity mean values in Qbox".(**S10 Table)**

### Identification of reliability criteria for 2D-SWE measurements

We retrieved five definitions of failure and ten definitions of non-reliability. (S2 **Table**). We were able to assess four criteria of quality.

For the "minimal 2D-SWE value" we confirmed a discriminant cut-off at 0.2 kPa. The LCC was not significant between 2D-SWE and FibroTest, only in patients with minimal elasticity <0.2kPa (**Table 1**)**(S4 Table) (S1 and S2 Figs).**

**Table 1:** Comparison of SWE minimal elasticity value cut-offs according to elasticity concordance with the 3 other reliable tests' results (FibroTest, TE-M, TE-XL), in the "Reliability population" (n = 1,720).

	Patients’ groups according to minimal elasticity value reported by 2D-SWE (range: 0-300kPa)							
Cutoff (kPa)	<0.2 kPa		0.2–0.5 kPa		0.5–1.0 kPa		> = 1 kPa	
n	132		221		209		1158	
	LCC mean	95% CI	LCC	95% CI	LCC	95% CI	LCC	95% CI
FibroTest	0.028^**1**^	-0.119;0.175	0.291	0.204;0.373	0.257	0.173;0.337	0.276	0.242;0.310
TE-M	0.287	0.140;0.421	0.658	0.583;0.722	0.674	0.595;0.740	0.724	0.697;0.749
TE-XL	0.289	0.133;0.431	0.654	0.574;0.722	0.562	0.464;0.647	0.640	0.606;0.671

LCC: Lin Concordance Coefficient.

^1^ No significant LLC between FibroTest and 2D-SWE, in patients with minimal elasticity <0.2kPa, as observed in a previous study.

In patients with values above the pre-determined cutoff (> = 0.2kPa), 2D-SWE was significantly associated (P-value <0.0001) with FibroTest. 2D-SWE elasticity values were also significantly more concordant with TE-M and TE-XL values than in patients with 2D-SWE minimal elasticity value <0.2 kPa (P<0.001).

For the elasticity CV (**S10 Table**), for elasticity measure depth **(S11 Table)**, and for BMI **(S12 Table**), we did not found sufficiently discriminant cutoffs comparatively to the "minimal 2D-SWE value".

### Applicability rates

Applicability of 2D-SWE was 89.6% (88.2–90.8), greater than that of TE-M (85.6% (84.0–87.0;P<0.0001), not different than TE-XL 88.2% (86.8–89.5;P = 0.15) and lower than FibroTest 99.5% (99.1–99.8;P<0.0001). TE-M applicability was lower than TE-XL (P = 0.008).(**Table 2**). In a total of 145 patients, elasticity measure was applicable using 2D-SWE and not applicable using TE-M.(**S6 Table)** In a total of 53 patients, elasticity measure was applicable using 2D-SWE despite not applicable using TE-XL.(**S7 and S8 Tables**). These patients who benefit from 2D-SWE had a higher prevalence of NAFLD.

**Table 2:** Applicability of fibrosis tests in investigated patients.

Biomarkers	2D-SWE		TE-M		TE-XL		FibroTest	
	n	% (95%CI)	n	% (95%CI)	n	% (95%CI)	n	% (95%CI)
Investigated	2,251	100	2,251	100	2,251	2,251	2,251	2,251
**Applicable**	**2,016**	**89.6 (88.2–90.8)**^**1**^	**1,926**	**85.6 (84.0–87.0)** ^**2**^	**1,986**	**88.2 (86.8–89.5)**	**2,239**	**99.5 (99.1–99.8)**
Not applicable	235	10.4 (9.2–11.7)	325	14.4 (13.0–16.0)	265	11.8 (10.5–13.2)	12	0.5 (0.1–0.9)
Failure	21	0.9	184	8.2	63	2.8	0	0.0
Not reliable	214	9.5	141	6.2	202	9.0	12	0.5

^1^Applicability of 2D-SWE was greater than that of TE-M (Z-test = 4.1;P<0.0001), not different than TE-XL (Z-test = 1.4;P = 0.15) and lower than FibroTest (Z-test = -14.6;P<0.0001).

^**2**^TE-M applicability was lower than TE-XL (Z-test = -2.7;P = 0.008). TE-M and TE-XL applicability were both lower than that of FibroTest (P<0.0001).

Among the 214 non-reliable SWE of this table, only 132 were identified in the SWE reliability population (132/1720 = 7.7%) using the minimal value cutoff 0.2 kPa. The remaining 82 patients had also not reliable TE- or TE-XL and were excluded of the intention to diagnose population (Fig 1).

### Curves fitting

Firstly, curves fitting identified linear regression in three parts as the best model for assessing the association between the elasticity values and fibrosis presumed by FibroTest **(S3 Fig)**, and whatever the liver disease **(S4 Fig)**.

Without stratification on fibrosis severity, 2D-SWE elasticity was less impacted by inflammation (**S5 Fig**) than TE-M and TE-XL, and whatever the liver disease **(S6 Fig)**. 2D-SWE elasticity was less impacted by steatosis **(S7 Fig)** than TE-M, but not than TE-XL, and whatever the liver disease (**S8 Fig**). For 2D-SWE, R2 = 0.07 lower than that of TE-M (0.12;P<0.01). Curves fitting using SteatoTest **(S7 Fig)** or CAP **(S9 Fig)** were similar.

#### Impact of inflammation and steatosis on elasticity values stratified by fibrosis severity (Fig 3)

**Fig 3:**
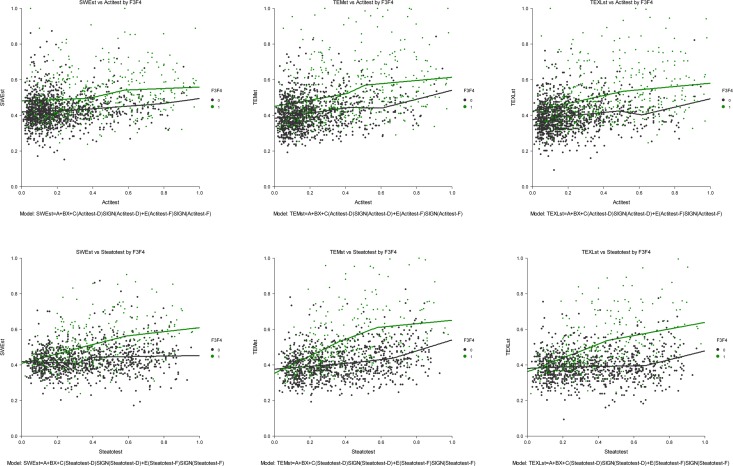
Elasticity values according to inflammation, or steatosis, among patients with or without severe fibrosis (F3F4). (A) 2D-SWE elasticity according to inflammation. (B) TE-M elasticity according to inflammation. (C) TE-XL elasticity according to inflammation. (D) 2D-SWE elasticity according to steatosis. (E) TE-M elasticity according to steatosis. (F) TE-XL elasticity according to steatosis. If not impacted by inflammation or steatosis the curves should be flat. 2D-SWE was less impacted than TE-M.

2D-SWE (**Fig 3 Panel A**) was less impacted by inflammation than TE-M (**Fig 3 Panel B**) and TE-XL (**Fig 3 Panel C**). In patients F0F1F2 the 2D-SWE R2 was 0.04, lower than that of TE-M (0.09; P<0.05) and not different than TE-XL (0.02; P>0.05) respectively. In patients F3F4 the 2D-SWE R2 = 0.17, lower than those of TE-M (0.24) and TE-XL (0.21) respectively (P<0.01).

2D-SWE (**Fig 3 panel D**) was less impacted by steatosis than TE-M (**Fig 3 Panel E**), but not than TE-XL (**Fig 3 Panel F**). In patients F0F1F2 the 2D-SWE the R2 was 0.04, lower than that of TE-M (0.09; P<0.05) and not different than TE-XL (0.02; P>0.05), respectively. In patients F3F4 the 2D-SWE the R2 was 0.17, lower than those of TE-M (0.24) and TE-XL (0.21) respectively (all P<0.01).

### Quantitative concordances (Table 3)

#### Elasticity and fibrosis

Without adjustments, LCC was lower for 2D-SWE vs TE-M and TE-XL, 0.298(0.250–0.309) vs 0.393(0.361–0.423) and vs 0.370(0.347–0.407) respectively (All P<0.01). After stratification by diseases, LCC remained significantly lower for 2D-SWE, only in patients with NAFLD.

**Table 3:** Quantitative concordance between elasticity values and severity of fibrosis, adjusted by inflammation, and steatosis. The strength of concordance was assessed by the Lin concordance correlation coefficient. Fibrosis, Steatosis and Inflammation were assessed by FibroTest, ActiTest and SteatoTest respectively.

Elasticity value concordance correlation coefficient: mean (95% CI)			
	Elastography method		
Correlation	2D-SWE	TE-M	TE-XL
**With fibrosis**			
All patients n = 1,588	0.280 (0.250–0.309)	0.393 (0.361–0.423)	0.370 (0.347–0.407)
**Adjusted by disease**^**1**^			
CHC n = 599	0.286 (0.235;0.334)	0.375 (0.314;0.433)	0.314 (0.263;0.363)
CHB n = 366	0.184 (0.132;0.235)	0.258 (0.196;0.318)	0.245 (0.186;0.302)
NAFLD n = 404	0.236 (0.176;0.295)	0.378 (0.316;0.436)	0.410 (0.347;0.469)
ALD n = 75	0.389 (0.246;0.507)	0.539 (0.408;0.648)	0.511 (0.379;0.622)
**Adjusted by significant inflammation**			
A0A1 n = 1,372	0.213 (0.180;0.245)	0.302 (0.267;0.336)	0.300 (0.257;0.333)
A2A3 n = 216	0.295 (0.213;0.373)	0.441 (0.350;0.524)	0.405 (0.321;0.482)
**Adjusted by significant steatosis**			
S0S1 n = 997	0.239 (0.201;0.275)	0.349 (0.310;0.388)	0.309 (0.270;0.347)
S2S3S4 n = 273	0.375 (0.307;0.440)	0.478 (0.412;0.539)	0.524 (0.458;0.584)
**Adjusted by inflammation and steatosis**			
A0A1-S0S1 n = 894	0.177 (0.138;0.216)	0.274 (0.232;0.316)	0.239 (0.197;0.280)
A2A3-S0S1 n = 103	0.298 (0.186;0.402)	0.418 (0.288;0.532)	0.373 (0.257;0.477)
A0A1-S2S3S4 n = 207	0.325 (0.249;0.396)	0.385 (0.308;0.457)	0.452 (0.373;0.524)
A2A3-S2S3S4 n = 66	0.322 (0.169;0.461)	0.495 (0.341;0.624)	0.514 (0.359;0.641)
**Adjusted on tests' interval**			
Less or 30 days n = 1535	0.283 (0.253–0.313)	0.397 (0.365–0.429)	0.385 (0.354–0.415)
More than 30days n = 53	0.182 (0.016;0.337)	0.260 (0.087–0.418)	0.156 (-0.019;0.332)
**With inflammation**			
All patients n = 1,588	0.135 (0.112–0.158)	0.220 (0.193–0.246)	0.203 (0.176–0.230)
**Adjusted by fibrosis severity**^**2**^			
F0F1F2 n = 1,198	0.039 (0.021;0.058)	0.090 (0.068;0.112)	0.063 (0.041;0.086)
F3F4 n = 390	0.146 (0.074;0.215)	0.236 (0.159;0.309)	0.228 (0.151;0.302)
**Adjusted on tests' interval**			
Less or 30 days n = 1535	0.135 (0.112–0.159)	0.223 (0.196–0.250)	0.207 (0.179–0.234)
More than 30days n = 53	0.112 (-0.007;0.228)	0.122 (-0.173;0.260)	0.156 (-0.019;0.332)
**With steatosis**			
All patients n = 1,270	0.167 (0.132;0.203)	0.263 (0.224;0.302)	0.175 (0.134;0.215)
**Adjusted by fibrosis severity**^**3**^			
F0F1F2 n = 989	0.105 (0.068;0.141)	0.192 (0.153;0.230)	0.074 (0.033;0.113)
F3F4 n = 281	0.286 (0.208;0.360)	0.369 (0.288;0.445)	0.357 (0.274;0.435)
**Adjusted on tests' interval**			
Less or 30 days n = 1252	0.169 (0.134;0.205)	0.265 (0.226;0.304)	0.178 (0.137;0.218)
More than 30days n = 18	0.043 (-0.216;0.297)	0.123 (-0.195;0.418)	-0.052 (-0.372;0.279)

^1^ After stratification by liver disease 2D-SWE elasticity remained less concordant with fibrosis severity presumed by FibroTest, only in patients with NAFLD, in comparison with TE-M and TE-XL

^2^Elasticity value estimated by 2D-SWE in patients with non-severe fibrosis was less correlated with significant inflammation presumed by ActiTest than TE-M (P<0.05).

^3^Elasticity value estimated by 2D-SWE in patients with non-severe fibrosis was less correlated with significant steatosis presumed by SteatoTest than TE-M (P<0.05). (n = 1270)

After stratification on inflammation severity, LCC remained significantly lower for 2D-SWE only in patients without significant inflammation, in comparison with TE-M and TE-XL. After stratification on steatosis severity, LCC remained significantly lower for 2D-SWE in patients without significant inflammation in comparison with TE-M, and in patients with significant steatosis in comparison with TE-XL.

After stratification on both inflammation and steatosis, LCC remained significantly lower for 2D-SWE, only in patients without significant inflammation/steatosis. The exclusion of patients (3%) with interval greater than 30 days did not change significantly the LCC for activity or steatosis the results.

#### Elasticity and inflammation

2D-SWE were less associated (LLC) with inflammation than TE-M, and TE-TXL: 0.135(0.112–0.158), 0.220(0.193–0.246), 0.203(0.176–0.230), respectively (all P<0.01). After stratification on severity of fibrosis, LCC remained significantly lower for 2D-SWE, than TE-M among patients without severe fibrosis.

#### Elasticity and steatosis

2D-SWE was less associated (LCC = 0.167[0.132;0.203]) with steatosis than TE-M (0.263 [0.224;0.302] P<0.01). After stratification on severity of fibrosis, LCC remained significantly lower for 2D-SWE vs TE-M. There was no significant difference between strength of concordance (LLC) of elasticity values estimated by 2D-SWE, TE-M or TE-XL, and BMI (P>0.05)**(S13 Table).**

#### Multivariate analyses

The multivariate analyses showed that taking into account the inflammation, the steatosis, and the cause of liver disease improved all the elasticities performances for the diagnosis of F3F4. The 2D-SWE AUROC was the most improved from 0.716(0.678;0.751) to 0.816(0.776;0.836;P<0.0001)**(S14 Table**), as compared to 0.747(0.701;0.769) to 0.824 (0.794;0.849;P<0.0001)(**S15 Table**) for TE-M, and 0.747(0.711;0.778) to 0.824 (0.794;0.850;P<0.0001)(**S16 Table**) for TE-XL. Most of the improvement was associated with the inflammation adjustment. Steatosis adjustment was significant only for TE-M.

#### Variability of presumed prevalence of severe fibrosis (F3F4) in CHC according to the combinations of elasticity results. (S17 Table)

Using FibroTest as a reference, the prevalence of F3F4 was 35.4%(31.6–39.4), higher than those presumed by 2D-SWE (19.0%;16.0–22.4), TE-M (19.2%;16.1–22.6) and TE-XL (15.9%;13.0–19.0)(All P<0.001).

Using the worst elasticity value among the 3 elasticity values, the presumed prevalence of F3F4 was 27.4%(23.8–31.1), and using only patients with the three concordant values, the prevalence of F3F4 was only 10.5%(8.2–13.5)(P<0.0001).

#### Consequence of inflammation and steatosis on the prevalence of severe fibrosis, as presumed by elasticity methods in CHC

According to elastography method, inflammation and steatosis, the presumed prevalence of F3F4 varied from 9.6%(6.7–13.3) using TE-XL in patients without significant inflammation and without significant steatosis, to 72.0%(50.6–87.9; P<0.0001) using TE-M in patients with significant inflammation and significant steatosis.

#### Analyses of diagnostic performances in intention to diagnose

In intention to diagnose, when applicability and inflammation were taken into account, 2D-SWE had higher performance for the diagnosis of F3F4 versus TE-M, (0.780 [0.756;0.802]) vs (0.764[0.740;0.786];P = 0.01), difference which was not identified in standard per-protocol comparisons (0.719[0.686;0.749] vs 0.739[0.708;0.767] P = 0.09) **(S18 Table)**.

## Discussion

An ideal study would have been to obtain a large surgical biopsy for each patient included, a perfect reference. In the absence of such perfect references, the standard method was to use biopsies, a non-perfect reference, with its own limitations including sampling error. Even 25 mm length biopsies had 25% of false positive/negative rates for the diagnosis of fibrosis stage, activity grade and steatosis grade [20,21]. The third approach we choose was to use validated blood tests as non-perfect references, with their own limitations and advantages. These approaches were complementary, and could permit to respond more rapidly to unmet needs [4, 22].

### Limitations of blood tests as references

The first main concern was that the reference utilized to assess fibrosis, inflammation and steatosis were suboptimal. We acknowledge that FibroTest had limitations, but had been extensively and independently validated with a low risk of non-reliable results, around 2% [11]. Even if the discordances rates were always around 20% versus elasticity measurements or biopsy fibrosis score, the prognostic performances of FibroTest were similar or greater than those of biopsy or TE-M for the most frequent chronic liver diseases [2,23,24,25,26]. Furthermore, the natural history of fibrosis progression estimated using FibroTest was similar to that estimated using biopsy [27]. Liver fibrosis progression was assessed using biopsy and FibroTest in 2,472 untreated patients: 770 with CHC, 723 with CHB, 761 with NAFLD, and 218 with ALD. We observed highly significant concordance between FibroTest and biopsy estimates of hazards with intraclass correlation = 0.961 (95% CI 0.948–0.970) and 0.899 (95% CI 0.135–0.969) for cirrhosis and for minimal fibrosis, respectively. This concordance persisted according to the disease and the gender.

ActiTest also has been extensively histologically validated (5,326 patients) and is the only blood tests with diagnostic performance greater than transaminases for the prediction of necro-inflammatory histologic activity [12,26]. Validation studies were not easily identified in PubMed (**S3 File).** One example of omitted evidence based was the largest histological validation of ActiTest in 1,459 patients of a prospective trial in CHC with biopsies [25].

We acknowledge that SteatoTest had much less studies available (3,253 patients), than FibroTest and ActiTest [13,26]. Validation studies were not easily identified in PubMed (**S4 File).** One example of omitted evidence based was the largest validation of SteatoTest in 1,415 patients of a prospective trial in CHC with biopsies [25].

The following other limitations were detailed in **S5 File.** We acknowledge that despite statistical significance, the difference in curve-fitting between TE and 2D-SWE were moderate and could be viewed as not clinically relevant. The measurements were not blinded as the same operator performed successively TE-M, TE-XL and 2D-SWE. We utilized the same cut-off values for 2D-SWE and TE-XL as of TE-M in the absence of consensual cut-offs. There was a low prevalence of patients with decompensated cirrhosis as one previously observed advantage of 2D-SWE was its higher applicability in patients with ascites than for TE-M.[4] We did not estimate automatic variability assessment, such as that combining CV and temporal variability.[5] Few patients (3%) had an interval between blood tests and elasticity measurements between one to six months but their exclusion did not change significantly the results.(**Table 3**) There was missing data for SteatoTest in 318 patients, but these patients were similar to those with non-missing SteatoTest.

### Advantages of the present study

#### Standardization of elasticity measures

The first original result was that logarithmic transformation but also standardization according to range had a direct impact for concordance analyses. 2D-SWE has a possible range up to 300kPa compared to a 75kPa maximum value for TE-M. A method with larger range of elasticity values will have an artificial decrease in quantitative concordance coefficient if not standardized as the reference method. These rules should be discussed in specific guidelines.

#### Quality criteria for 2D-SWE results

The second original result was the clarification of the relative interest of four quality criteria proposed for 2D-SWE. Among these criteria only the minimal value of the elasticity in the ROI minimal (0.2kPa) seemed useful as a cutoff to identify and exclude unreliable results. As this cutoff was predetermined and validated in a previous study, it could be recommended as a simple criterion for clinicians.[4]

#### Applicability of 2D-SWE

The better applicability rate of 2D-SWE versus TE-M was confirmed.[2,4] These patients who benefit from 2D-SWE had a higher prevalence of NAFLD. These results were in accordance with the lower impact of steatosis on elasticity measured by 2D-SWE. These differences confirmed that comparisons between tests must be performed in intention to diagnose [18].

#### Impact of inflammation and steatosis on 2D-SWE elasticity estimates

For the first time to our knowledge it was possible, in a large number of patients with different liver diseases, to assess the relative impact of inflammation and steatosis on the elasticity value, independently of fibrosis severity. The influence of inflammation on elasticity measured by TE-M has been well validated, but few studies were published for 2D-SWE.[2,11,12] The influence of steatosis on elasticity was a matter of debate with conflicting results in TE-M studies: some studies suggested that steatosis was associated to an increase in whereas did not.[2,28,29] Here we observed that for all elastography methods, elasticity was increased by both inflammation and steatosis. The curve fitting, the univariate and the multivariate analyses clearly demonstrated that inflammation increased more the elasticity value than steatosis. Steatosis measure presumed by SteatoTest seemed more sensitive than when presumed by CAP.

#### Comparisons between elastography methods, in per-protocol and intention to diagnose

As already described, the comparison between blood tests and elastography performances should be performed using direct comparisons, and intention to diagnose analyses.[18] In intention to diagnose, when applicability and inflammation were taken into account, 2D-SWE had higher performance for the diagnosis of F3F4 versus TE-M, difference which was not identified in standard per-protocol comparisons **(S18 Table).** In patients with high risk of inflammation or steatosis, 2D-SWE had an advantage for being more applicable and more specific for staging fibrosis than the TE-M, the present standard elastography method. Further studies are necessary to compare other new elastography methods such as ARFI or other real time elastography [9,30].

In patients with CHC, according to the severity of inflammation and steatosis, and to the elastography method, the presumed prevalence of F3-F4 varied from 9.6% to 72.0%. This spectrum variability should be taken into account for the prioritization of reimbursement of DAA.

## Conclusion

Elasticity results 2D-SWE including minimal signal <0.2 kPa in the ROI should be considered as not reliable. 2D-SWE had a higher applicability than TE-M the reference elastography, with less impact of inflammation and steatosis especially in patients with non-advanced fibrosis, as presumed by blood tests.

## Supporting Information

S1 Fig Distribution of minimal elasticity values, expressed after standardization, among 132 patients with measurements lower than 0.2 kPa. (DOCX)

S2 Fig Regression curves according to the classes of "Minimal elasticity values", The 132 patients with minimal elasticity value (<0.2 kPa) had insignificant correlation with FibroTest, contrarily to higher minimal elasticity values (>0.2kPa) (DOCX)

S3 Fig Association between elasticity estimates and fibrosis severity as presumed by FibroTest. Curve fitting in all patients.
(DOCX)

S4 Fig Association between elasticity estimates and fibrosis severity as presumed by FibroTest. Curve fitting according to the 5 causes of liver disease.
(DOCX)

S5 Fig Association between elasticity estimates and significant inflammation presumed by ActiTest in all patients (n = 1,588). (DOCX)

S6 Fig Curve fitting of elasticity according to inflammation, among the five causes of liver disease. (DOCX)

S7 Fig Association between elasticity estimates and steatosis presumed by SteatoTest, in all patients (n = 1,270). (DOCX)

S8 Fig Curve fitting of elasticity according to steatosis, among the five causes of liver disease. (DOCX)

S9 Fig Association between elasticity estimates and steatosis presumed by CAP (n = 1,549) (DOCX)

S10 Fig Curve fitting using controlled attenuation parameter (CAP) for presumed steatosis, according to the five causes of liver disease. (DOCX)

S1 File Detailed methods concerning patients. (DOCX)

S2 File Detailed methods concerning biomarkers. (DOCX)

S3 File Review of ActiTest validations articles. (DOCX)

S4 File Review of SteatoTest validations articles. (DOCX)

S5 File Details of other limitations of the present study. (DOCX)

S1 Table Published definitions of failures and reliability of elasticity values assessed by 2D-SWE. (DOCX)

S2 Table List and definitions of estimates of elasticity, recorded by 2D-SWE, for each patient. (DOCX)

S3 Table Impact of elasticity standardization criteria on concordance analyses (Lin concordance coefficient) in "concordance population with SteatoTest (n = 1270). (DOCX)

S4 Table Curves fitting report according to "Minimal elasticity values". (DOCX)

S5 Table Results of elasticity estimates of elasticity by 2D-SWE, which were recorded for each patient. (DOCX)

S6 Table Characteristics of patients with at least one not-applicable tests (2D-SWE, TE-M, TE-XL and FT) compared to patients with the four tests applicable (concordance population). (DOCX)

S7 Table Characteristics of patients included in the "concordance population without ST" (1,588–1,270 = 318) compared to the "concordance population" with ST (n = 1,270). (DOCX)

S8 Table Characteristics of patients with TE-M not-applicable tests and 2D-SWE applicable compared to patients of the "concordance population" (n = 1,588). (DOCX)

S9 Table Characteristics of patients with TE-XL not-applicable tests and 2D-SWE applicable compared to patients of the "concordance population" (n = 1,588). (DOCX)

S10 Table Comparison of the SWE coefficient of variation (ratio standard deviation/stiffness mean) cutoffs, according to concordance with the 3 other reliable tests' results. Reliability population, n = 1720.
(DOCX)

S11 Table Comparison of the SWE depth cutoffs, according to concordance with the 3 other reliable tests' results, in the "Reliability population" (n = 1,720). Depth (mm) measured from probe surface to the top of the region of interest.
(DOCX)

S12 Table Comparison of the WHO BMI cutoffs, according to concordance of SWE with the 3 other reliable tests' results, in the "reliability population" (n = 1,720). (DOCX)

S13 Table Association between elasticity values and body mass index according to elastography method, adjusted by fibrosis severity. (DOCX)

S14 Table Multivariate analysis of diagnostic performance of 2D-SWE elasticity for the diagnosis of F3F4 presumed by FibroTest, adjusted on inflammation, steatosis or liver disease. (DOCX)

S15 Table Multivariate analysis of diagnostic performance of TE-M elasticity for the diagnosis of F3F4 presumed by FibroTest, adjusted on inflammation, steatosis or liver disease. (DOCX)

S16 Table Multivariate analysis of diagnostic performance of TE-XL elasticity for the diagnosis of F3F4 presumed by FibroTest, adjusted on inflammation, steatosis or liver disease. (DOCX)

S17 Table Performances of elastography methods in CHC patients (n = 599) for the diagnosis of severe fibrosis (F3F4) presumed by FibroTest and using a standard cutoff of 9.5 kPa. Impact of inflammation and steatosis severities, presumed by ActiTest (n = 599) and SteatoTest (n = 477).
(DOCX)

S18 Table Comparison of diagnostic performances of SWE, TE-M, TE-XL for the diagnosis of severe fibrosis (F3-F4) presumed by FibroTest. Analyses performed in "Intention to diagnose" in 2,239 patients, and "Per protocol" in 1,588 patients with applicable elasticity data.
(DOCX)
