# Supplementary material for: Real-Time Shear Wave versus Transient Elastography for Predicting Fibrosis: Applicability, and Impact of Inflammation and Steatosis. A Non-Invasive Comparison
Source: PLoS One. 2016 Oct 5;11(10):e0163276. doi: 10.1371/journal.pone.0163276 (PMC5051706; doi:10.1371/journal.pone.0163276)
Supplement: S1 Fig — (DOCX) [file pone.0163276.s001.docx]

**S1 Fig. Distribution of minimal elasticity values, expressed after standardization, among 132 patients with measurements lower than 0.2 kPa.**
