# Supplementary material for: Real-Time Shear Wave versus Transient Elastography for Predicting Fibrosis: Applicability, and Impact of Inflammation and Steatosis. A Non-Invasive Comparison
Source: PLoS One. 2016 Oct 5;11(10):e0163276. doi: 10.1371/journal.pone.0163276 (PMC5051706; doi:10.1371/journal.pone.0163276)
Supplement: S2 Fig — (DOCX) [file pone.0163276.s002.docx]

**S2 Fig. Regression curves according to the classes of "Minimal elasticity values",**

The 132 patients with minimal elasticity value (<0.2 kPa) had insignificant correlation with FibroTest, contrarily to higher minimal elasticity values (>0.2kPa)

Classes of minimal elasticity value: 0 <0.2 kPa (Blue), 1: 0.2-0.5 kPa (green), 2: 0.5-1.0 kPa (black), and 3 >=1 kPa (orange)
