# Supplementary material for: Real-Time Shear Wave versus Transient Elastography for Predicting Fibrosis: Applicability, and Impact of Inflammation and Steatosis. A Non-Invasive Comparison
Source: PLoS One. 2016 Oct 5;11(10):e0163276. doi: 10.1371/journal.pone.0163276 (PMC5051706; doi:10.1371/journal.pone.0163276)

**S3 Fig. Association between elasticity estimates and fibrosis severity as presumed by FibroTest. Curve fitting in all patients.**

Curve fitting using regression modeling identified linear regression in 3 parts ("linear-linear-linear modeling") was the best model for assessing the association between the three elastography tests and fibrosis presumed by FibroTest.

The best model was a three linear model, with a squared correlation coefficient (R2) of 0.21, 0.33, and 0.32 for 2D-SWE, TE-M and TE-XL respectively, versus the following R2 with the simple linear modeling: 0.17, 0.25 and 0.25 respectively, all P<0.0001. This "linear-linear-linear model" was therefore applied for all the curves fitting


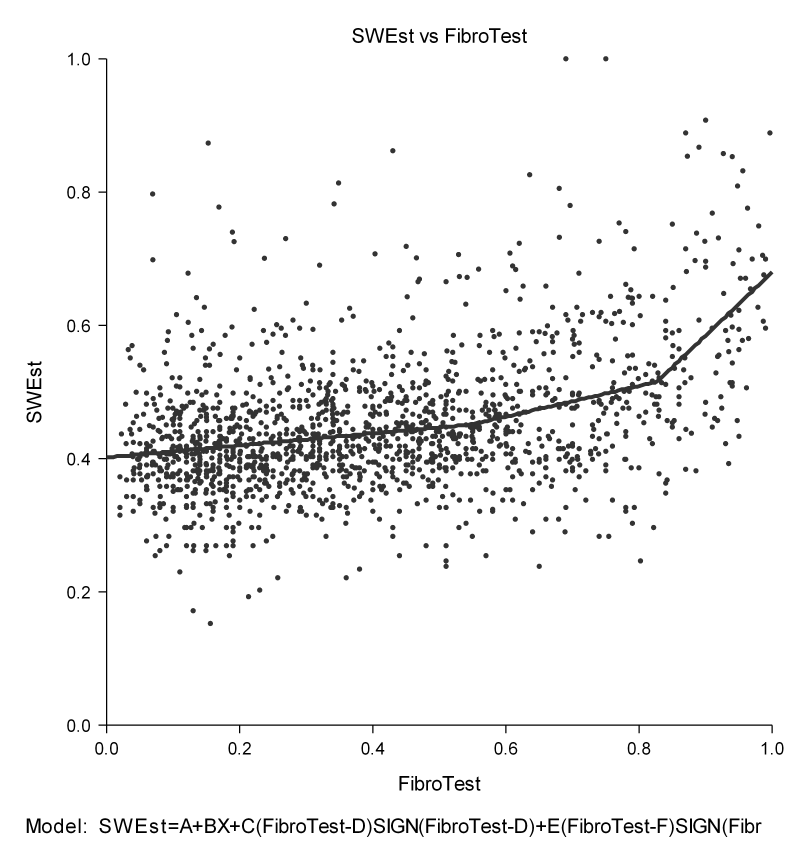

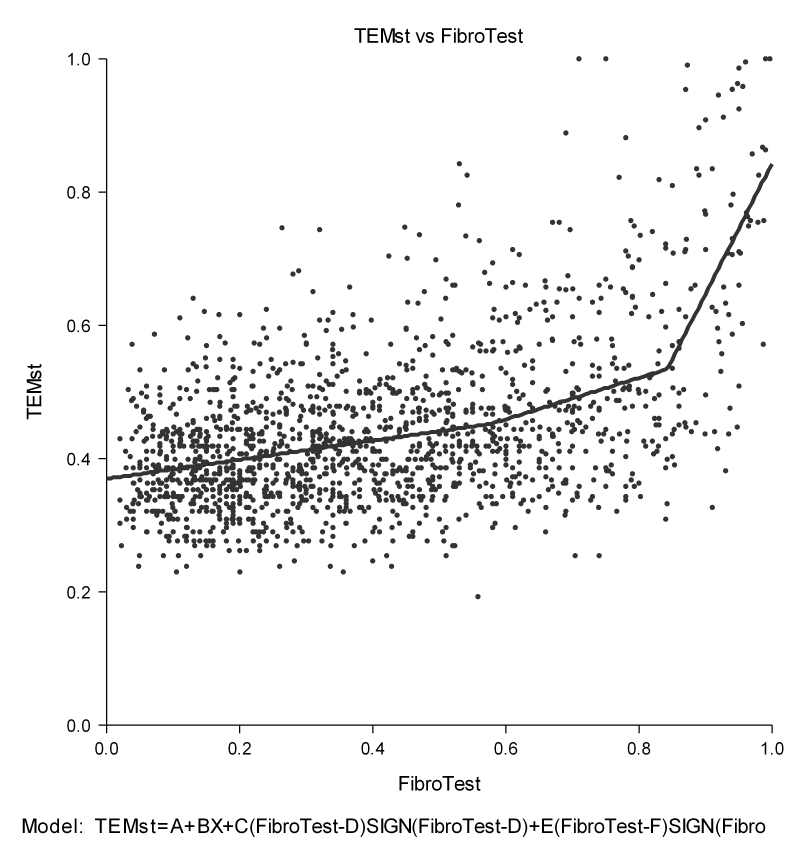

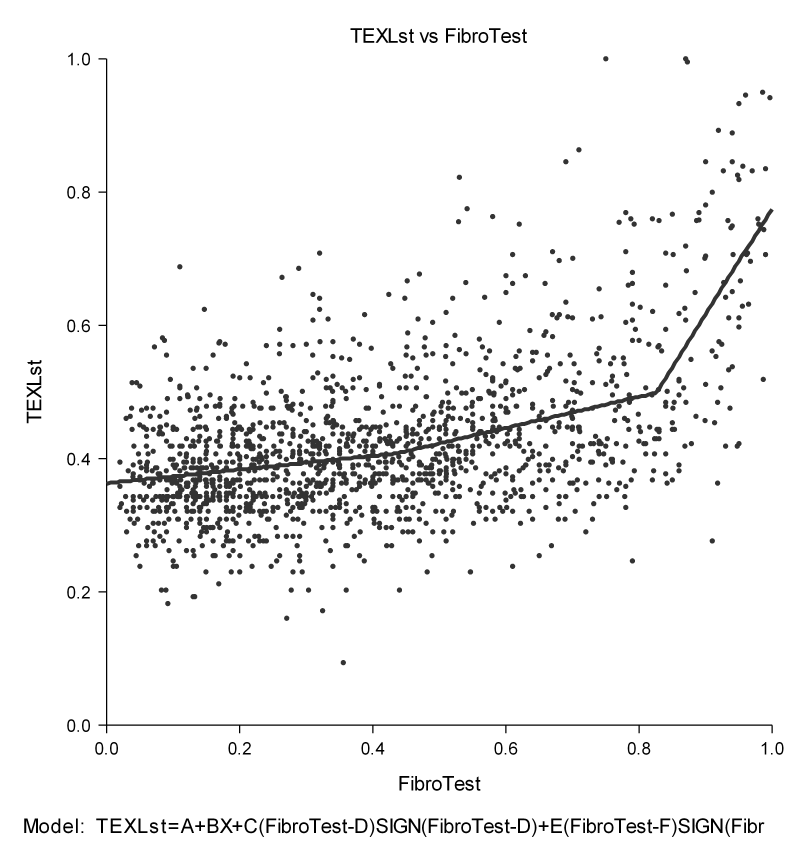

Supplement: S3 Fig — Curve fitting in all patients. (DOCX) [file pone.0163276.s003.docx]
