# Supplementary material for: Real-Time Shear Wave versus Transient Elastography for Predicting Fibrosis: Applicability, and Impact of Inflammation and Steatosis. A Non-Invasive Comparison
Source: PLoS One. 2016 Oct 5;11(10):e0163276. doi: 10.1371/journal.pone.0163276 (PMC5051706; doi:10.1371/journal.pone.0163276)

**S4 Fig. Association between elasticity estimates and fibrosis severity as presumed by FibroTest. Curve fitting according to the 5 causes of liver disease.**

According to linear-linear-linear model, R2 varied significantly according to liver disease for each test. For 2D-SWE from 0.16 (CHB) to 0.39 (ALD=0.21). Using TE-M, from 0.20 (CHB) to 0.51 (ALD), and for TE-XL from 0.22 (CHB) to ALD (0.48). All inequality tests P<0.0001.

According to linear-linear model, R2 varied significantly according to liver disease for each test. For 2D-SWE from 0.04 (CHC) to 0.08 (NAFLD). Using TE-M, from 0.03 (CHB) to 0.11 (ALD), and for TE-XL from 0.01 (CHC) to 0.08 (NAFLD). All inequality tests P<0.0001.


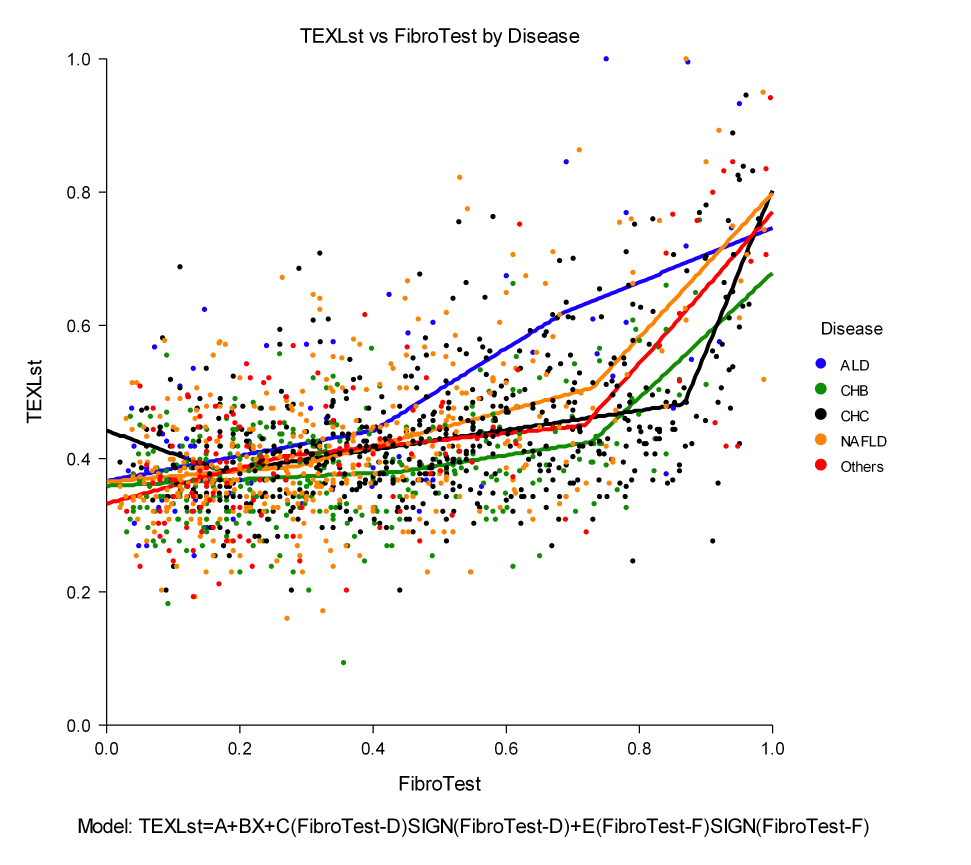

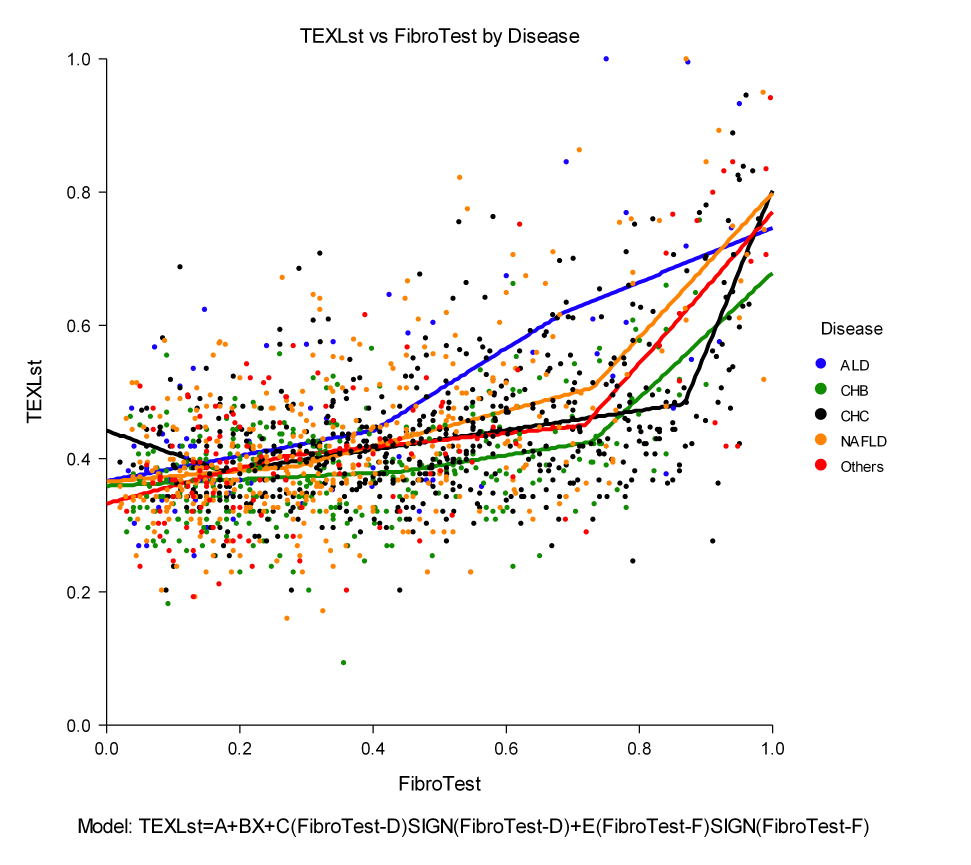

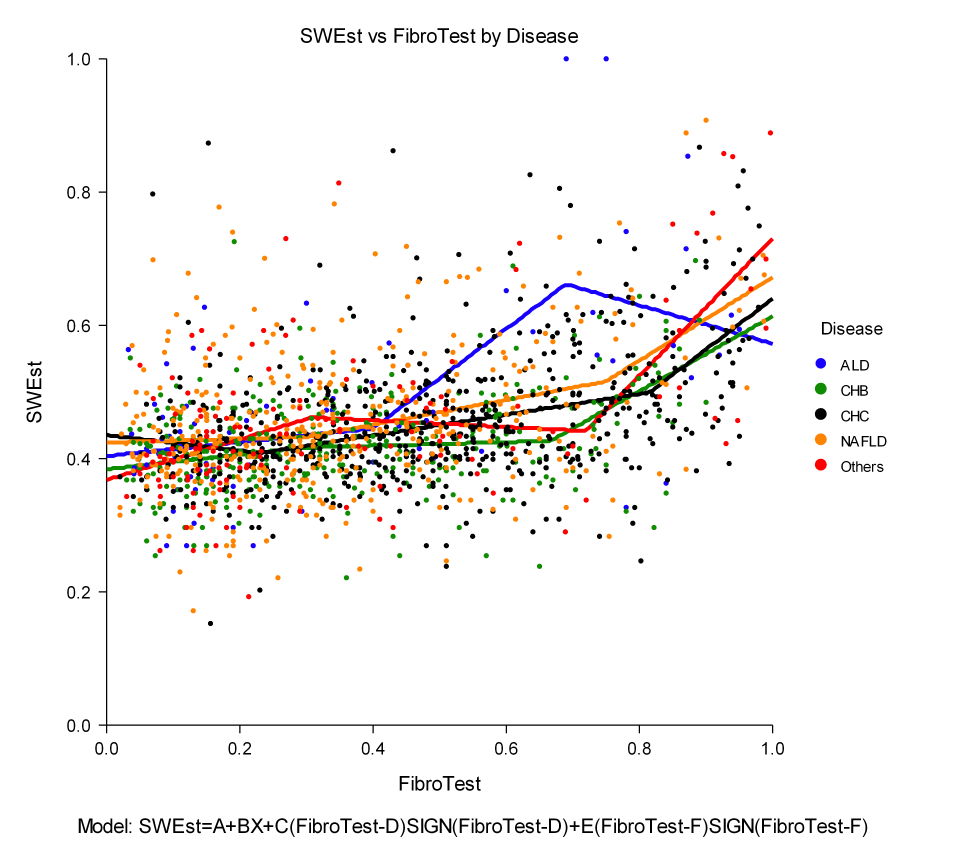

Supplement: S4 Fig — Curve fitting according to the 5 causes of liver disease. (DOCX) [file pone.0163276.s004.docx]
