# Supplementary material for: Real-Time Shear Wave versus Transient Elastography for Predicting Fibrosis: Applicability, and Impact of Inflammation and Steatosis. A Non-Invasive Comparison
Source: PLoS One. 2016 Oct 5;11(10):e0163276. doi: 10.1371/journal.pone.0163276 (PMC5051706; doi:10.1371/journal.pone.0163276)

**S5 Fig. Association between elasticity estimates and significant inflammation presumed by ActiTest in all patients (n=1,588).**

According to linear-linear-linear model, all R2 were significant (P<0.0001). R2 =0.08 for 2D-SWE, was lower than those of TE-M (0.16) and TE-XL (0.13), respectively (P<0.01).


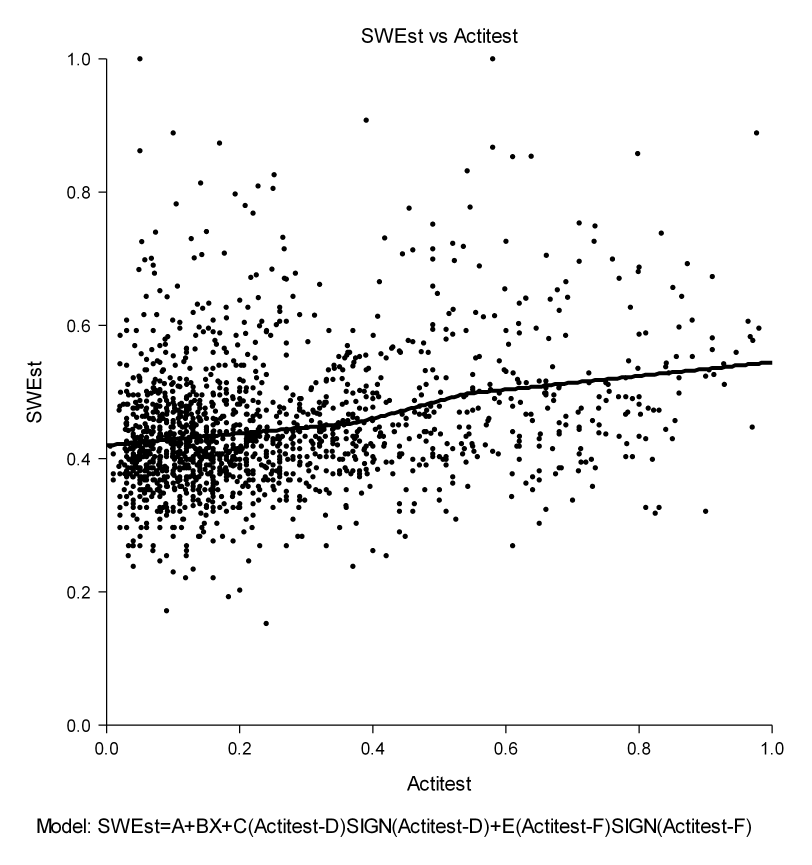

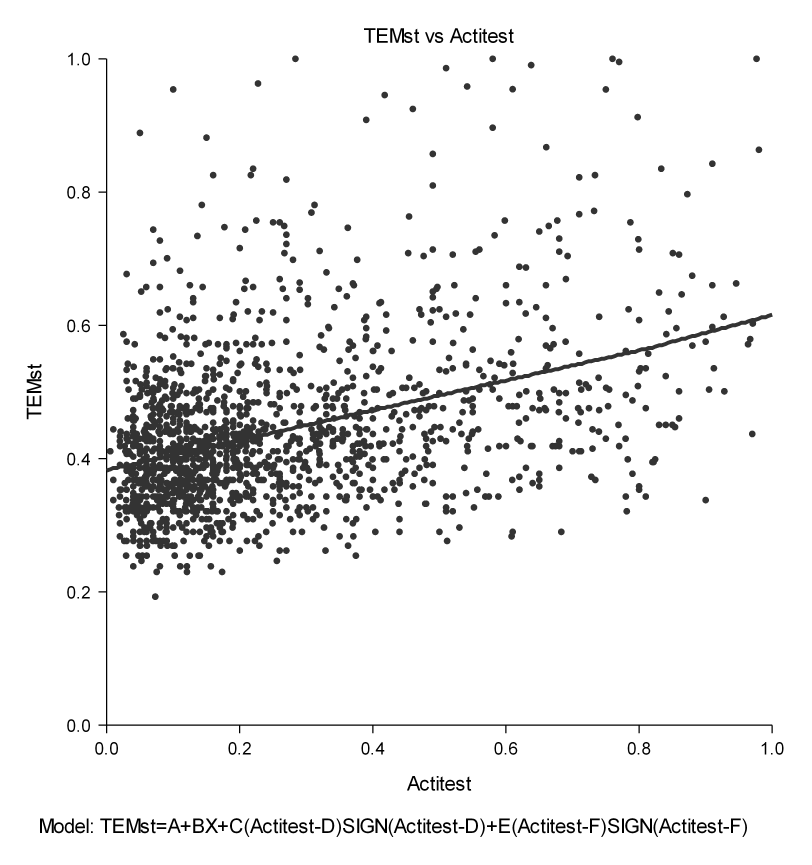

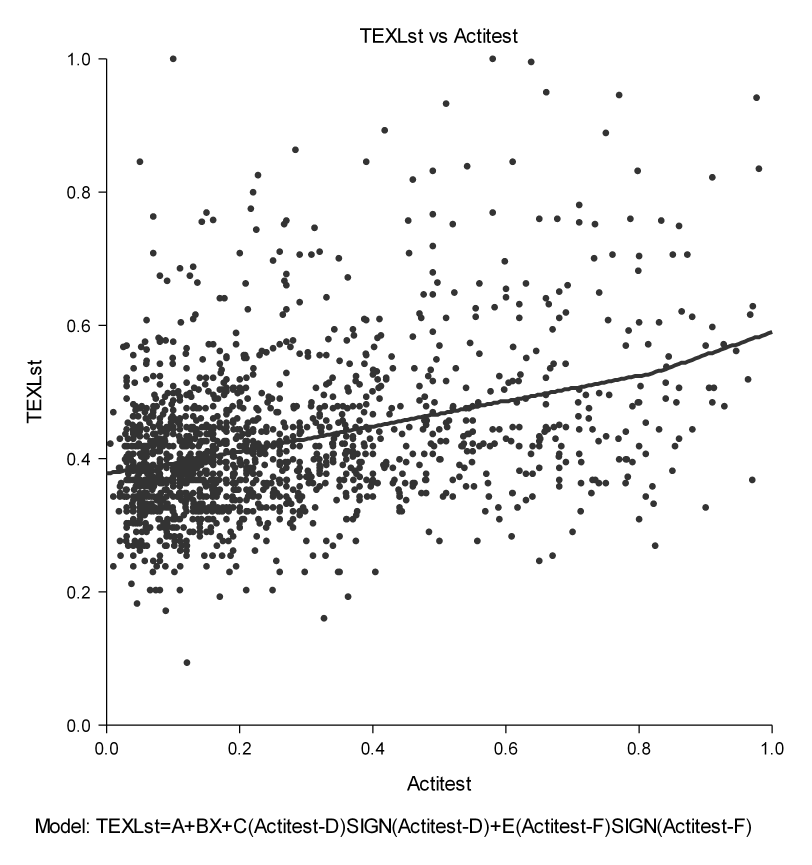

Supplement: S5 Fig — (DOCX) [file pone.0163276.s005.docx]
