# Supplementary material for: Real-Time Shear Wave versus Transient Elastography for Predicting Fibrosis: Applicability, and Impact of Inflammation and Steatosis. A Non-Invasive Comparison
Source: PLoS One. 2016 Oct 5;11(10):e0163276. doi: 10.1371/journal.pone.0163276 (PMC5051706; doi:10.1371/journal.pone.0163276)

**S6 Fig. Curve fitting of elasticity according to inflammation, among the five causes of liver disease.**

R2 varied significantly according to liver disease for each test. For 2D-SWE from 0.06 (NAFLD) to 0.13 (CHB). Using TE-M, from 0.13 (CHB) to 0.20 (ALD), and for TE-XL from 0.09 (CHB) to ALD (0.18). All inequality tests P<0.0001.


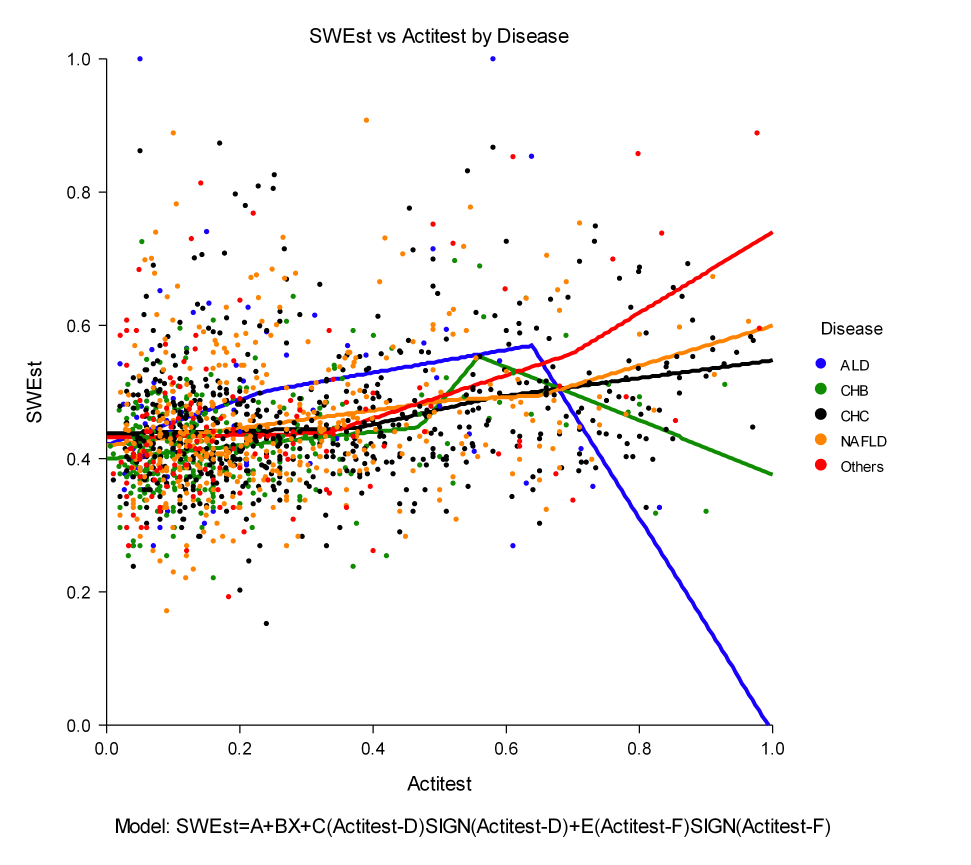

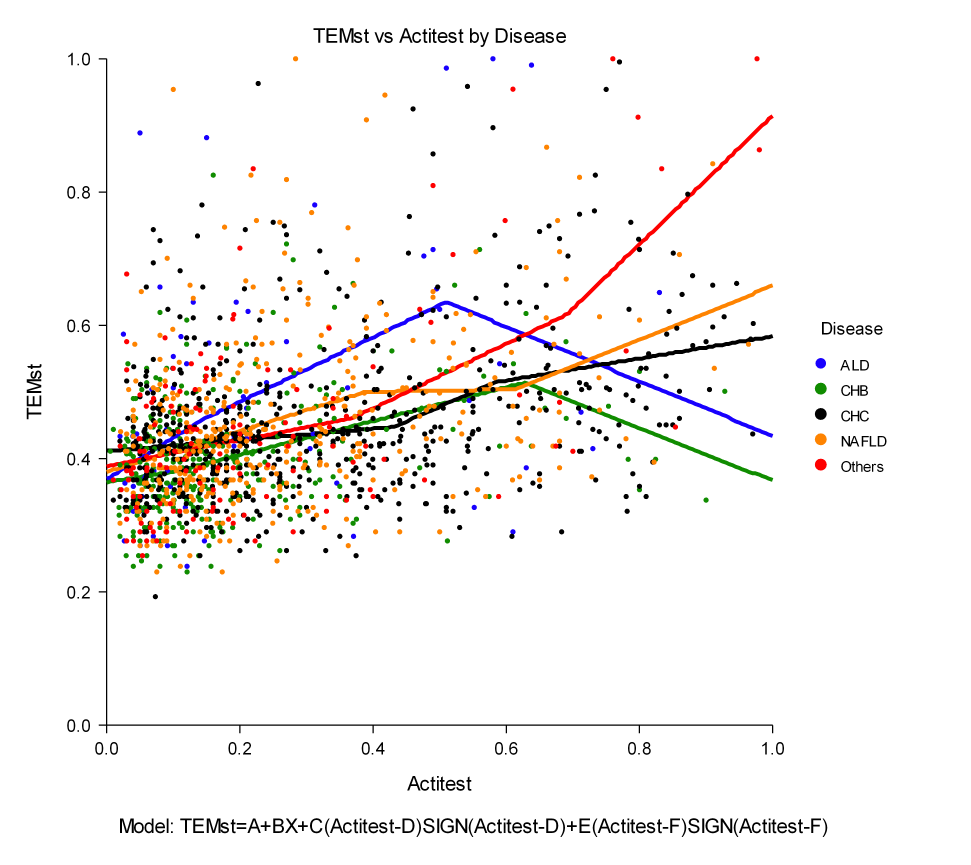

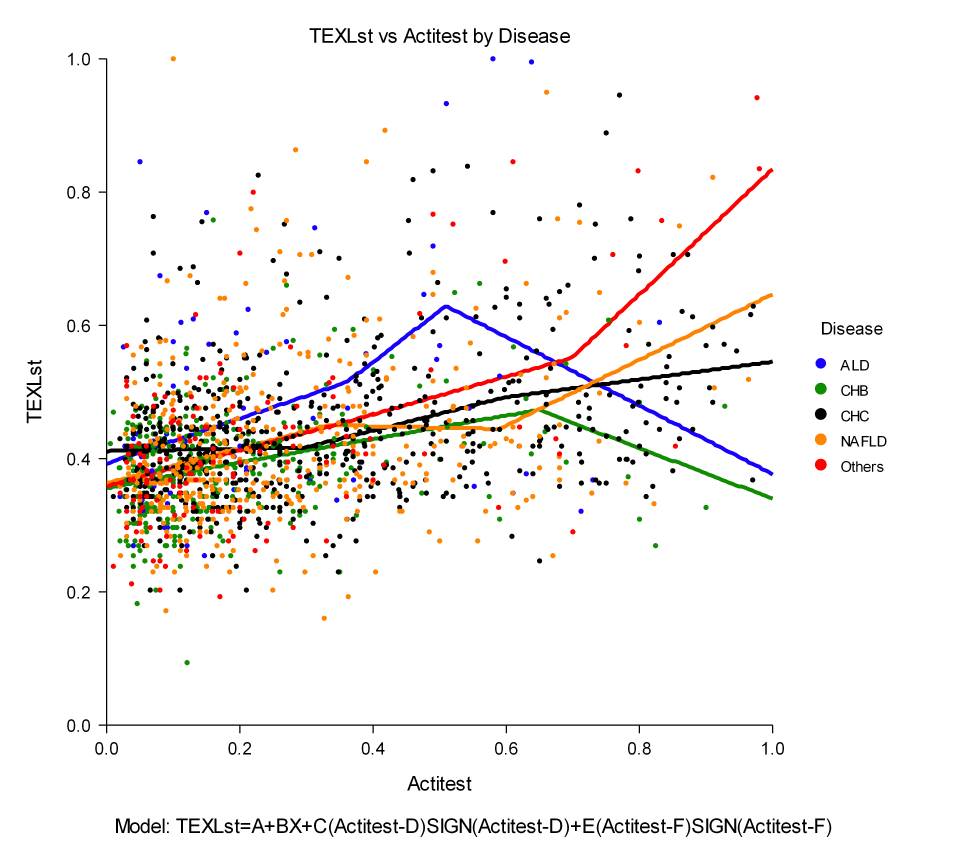

Supplement: S6 Fig — (DOCX) [file pone.0163276.s006.docx]
