# Supplementary material for: Real-Time Shear Wave versus Transient Elastography for Predicting Fibrosis: Applicability, and Impact of Inflammation and Steatosis. A Non-Invasive Comparison
Source: PLoS One. 2016 Oct 5;11(10):e0163276. doi: 10.1371/journal.pone.0163276 (PMC5051706; doi:10.1371/journal.pone.0163276)

**S7 Fig. Association between elasticity estimates and steatosis presumed by SteatoTest, in all patients (n=1,270).**


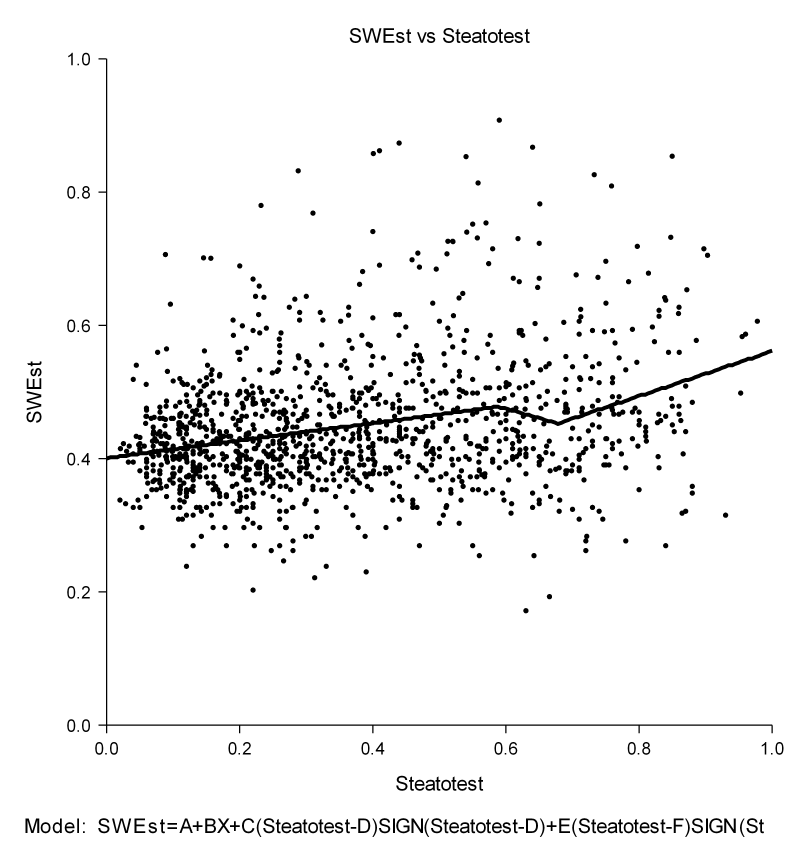

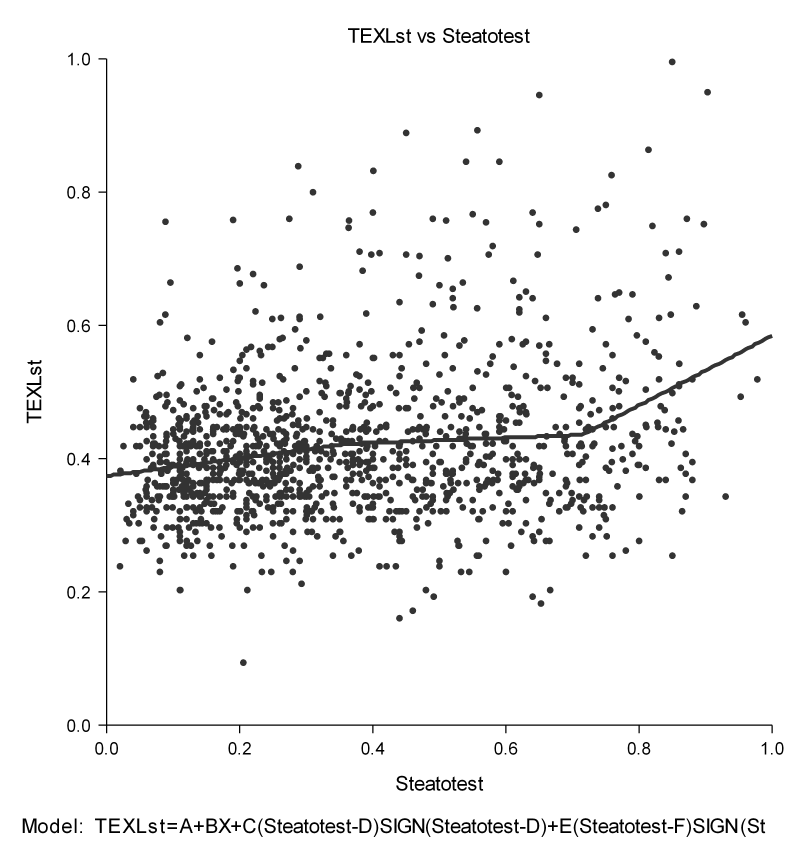

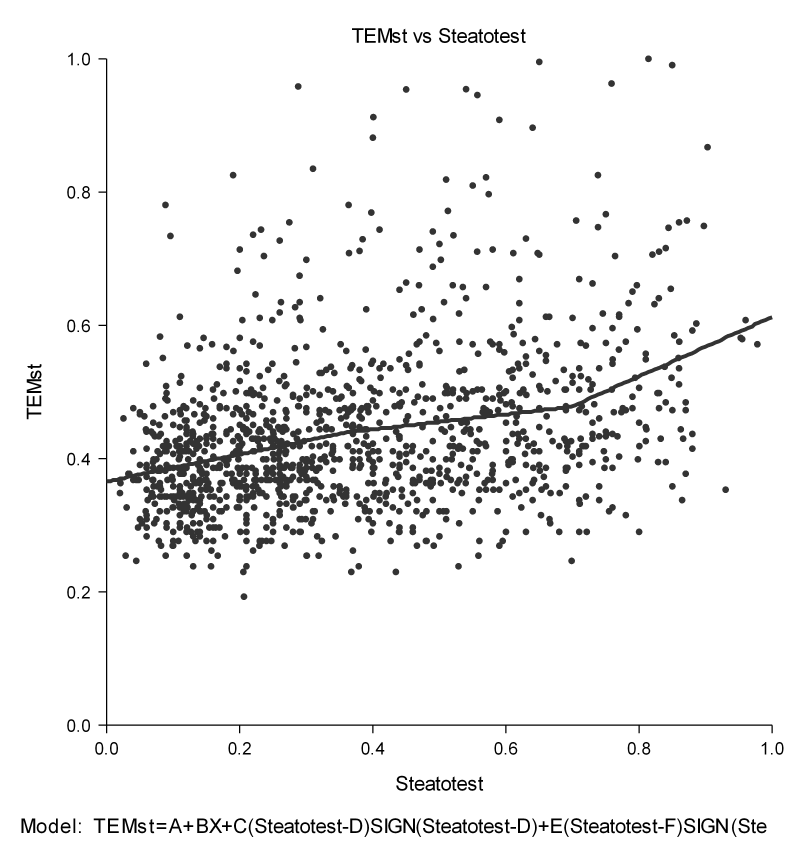

Supplement: S7 Fig — (DOCX) [file pone.0163276.s007.docx]
