# Supplementary material for: Real-Time Shear Wave versus Transient Elastography for Predicting Fibrosis: Applicability, and Impact of Inflammation and Steatosis. A Non-Invasive Comparison
Source: PLoS One. 2016 Oct 5;11(10):e0163276. doi: 10.1371/journal.pone.0163276 (PMC5051706; doi:10.1371/journal.pone.0163276)

**S8 Fig. Curve fitting of elasticity according to steatosis, among the five causes of liver disease.**

According to linear-linear-linear model, R2 varied significantly according to liver disease for each test. For 2D-SWE from 0.04 (CHB) to 0.13 (CHC), TE-M, from 0.05 (CHB) to 0.16 (CHC), and for TE-XL from 0.03 (CHB) to 0.11 (CHC). All inequality tests P<0.0001.


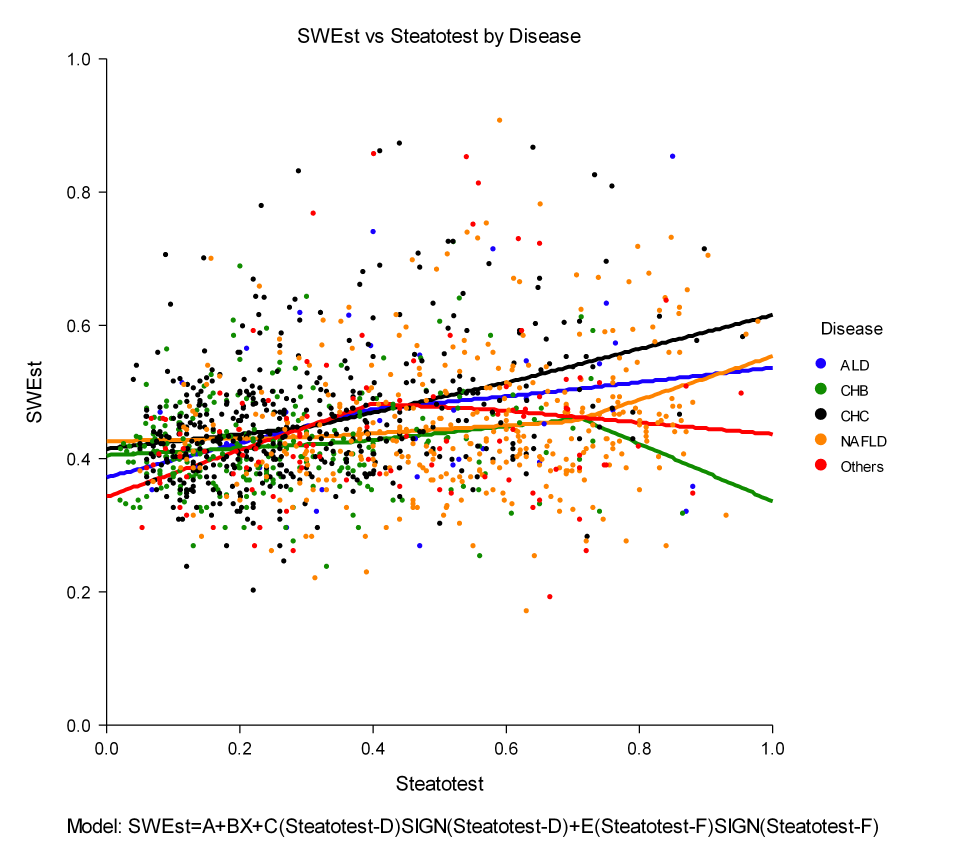

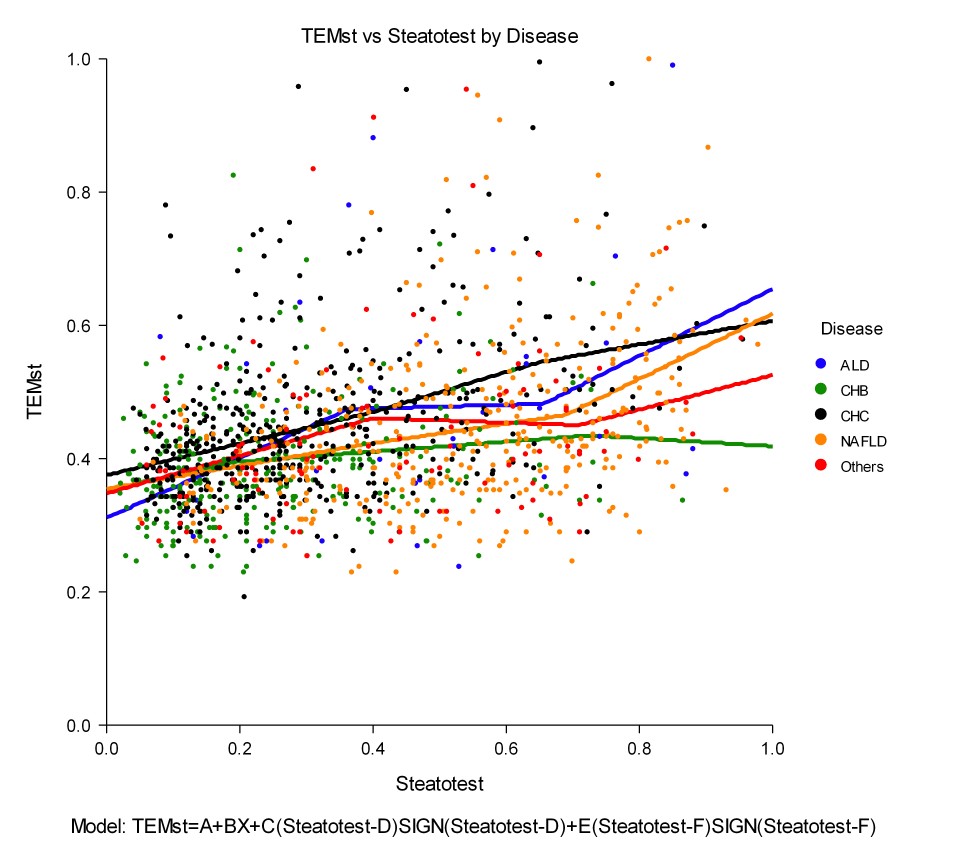

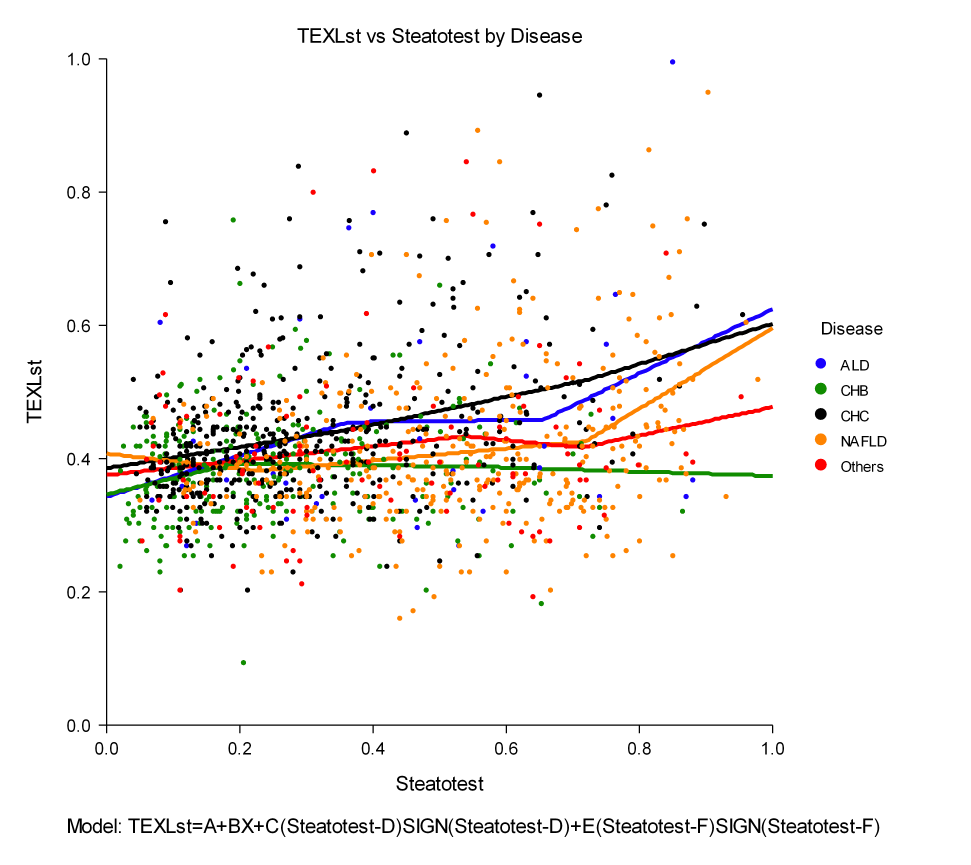

Supplement: S8 Fig — (DOCX) [file pone.0163276.s008.docx]
