# Supplementary material for: Real-Time Shear Wave versus Transient Elastography for Predicting Fibrosis: Applicability, and Impact of Inflammation and Steatosis. A Non-Invasive Comparison
Source: PLoS One. 2016 Oct 5;11(10):e0163276. doi: 10.1371/journal.pone.0163276 (PMC5051706; doi:10.1371/journal.pone.0163276)
Supplement: S9 Fig — (DOCX) [file pone.0163276.s009.docx]

**S9 Fig. Association between elasticity estimates and steatosis presumed by CAP (n=1,549)**


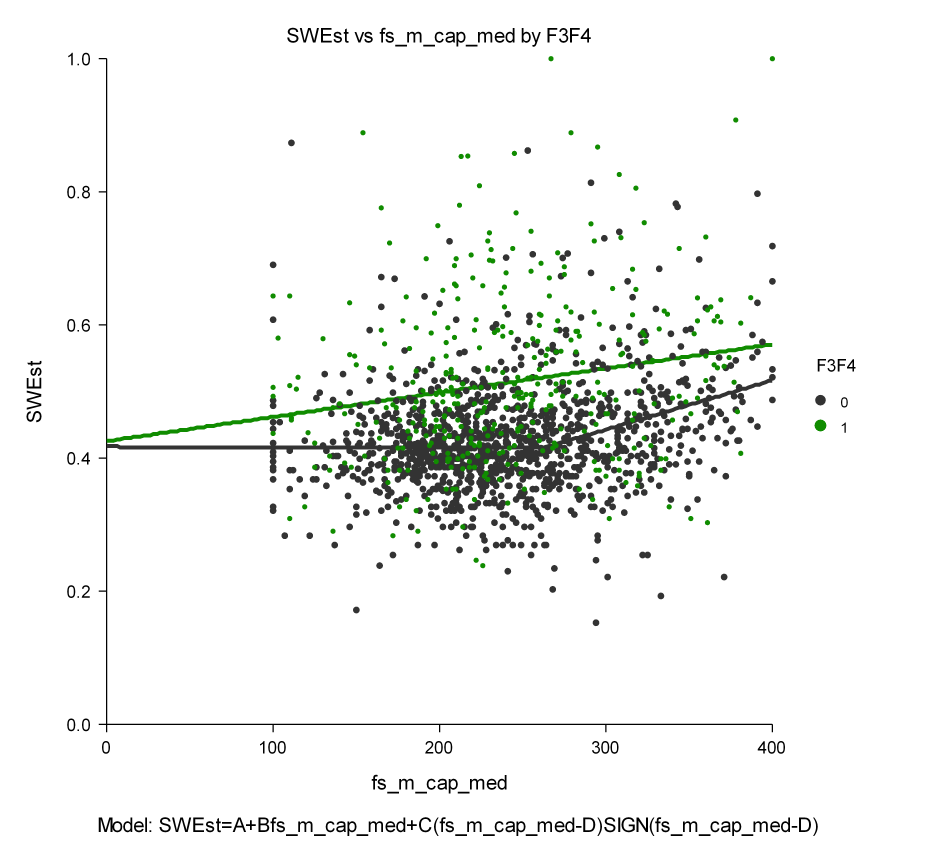

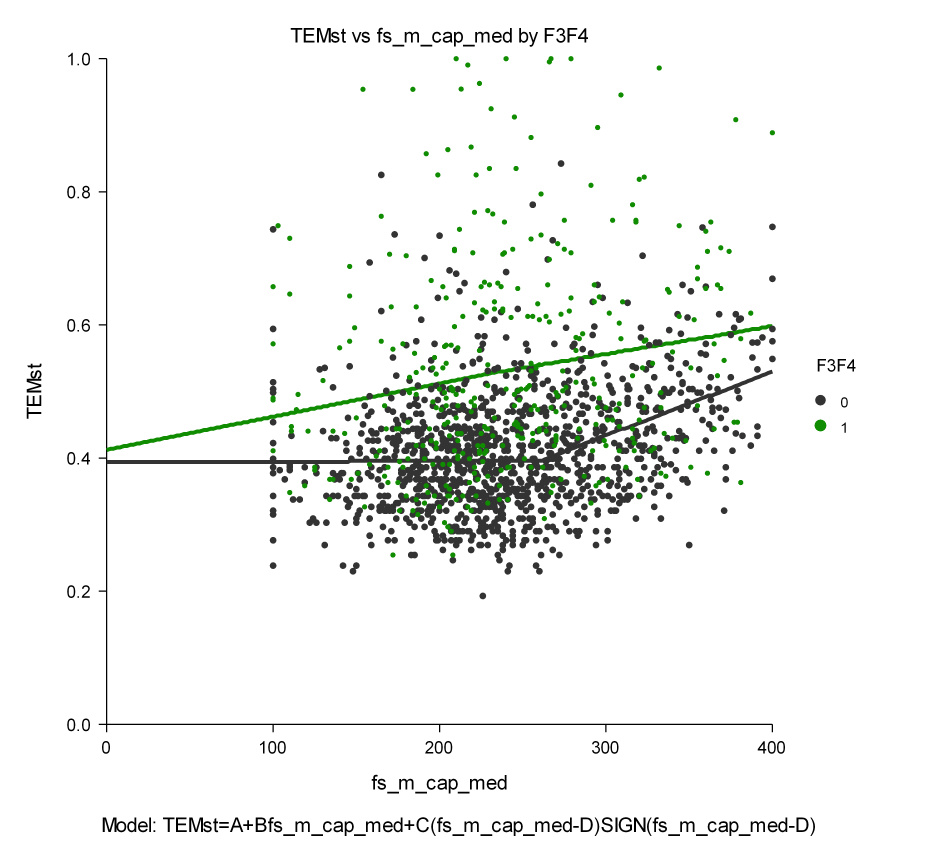

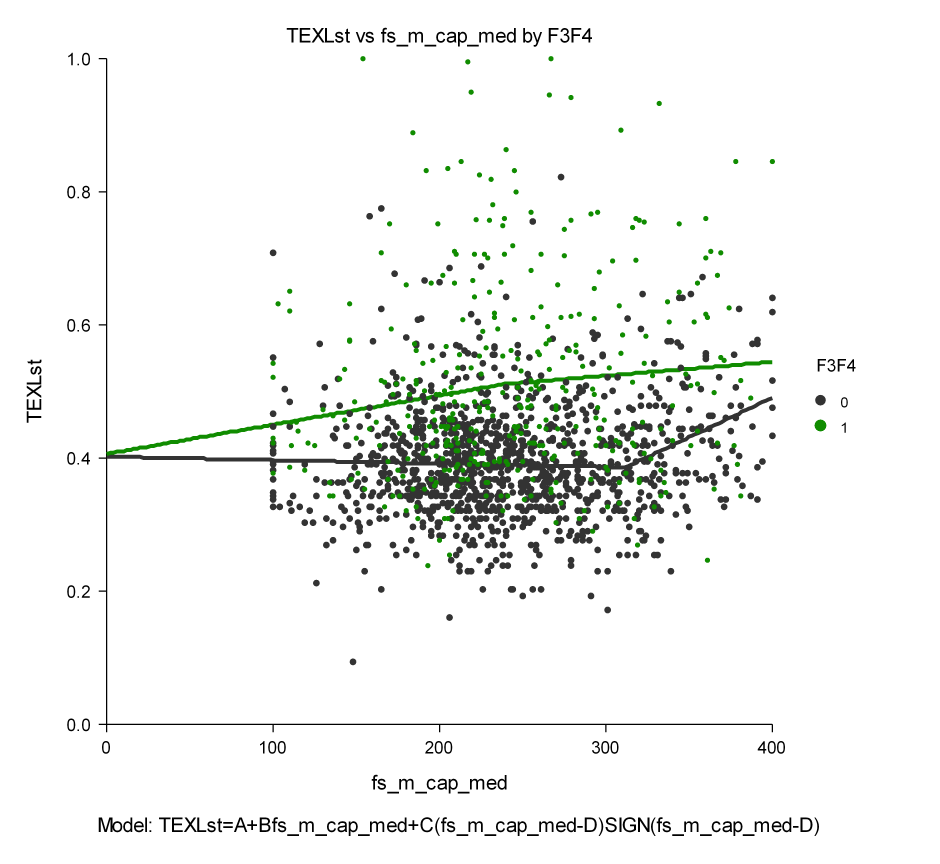


Curve fitting using CAP for presumed steatosis, among patients with or without severe fibrosis (F3F4), presumed by FibroTest.

Whatever the elastography method, patients F0F1F2 fitted better to a linear-linear model, in comparison with patients F3F4, with an increased slope above 200 dB/m (P<0.0001). In patients F0F1F2 the 2D-SWE the R2 was 0.07, lower than that of TE-M (0.11; P<0.05). In patients F3F4 there was no differences between elastography methods.
