# Supplementary material for: Real-Time Shear Wave versus Transient Elastography for Predicting Fibrosis: Applicability, and Impact of Inflammation and Steatosis. A Non-Invasive Comparison
Source: PLoS One. 2016 Oct 5;11(10):e0163276. doi: 10.1371/journal.pone.0163276 (PMC5051706; doi:10.1371/journal.pone.0163276)

**S10 Fig. Curve fitting using controlled attenuation parameter (CAP) for presumed steatosis, according to the five causes of liver disease.**

No differences were identified.


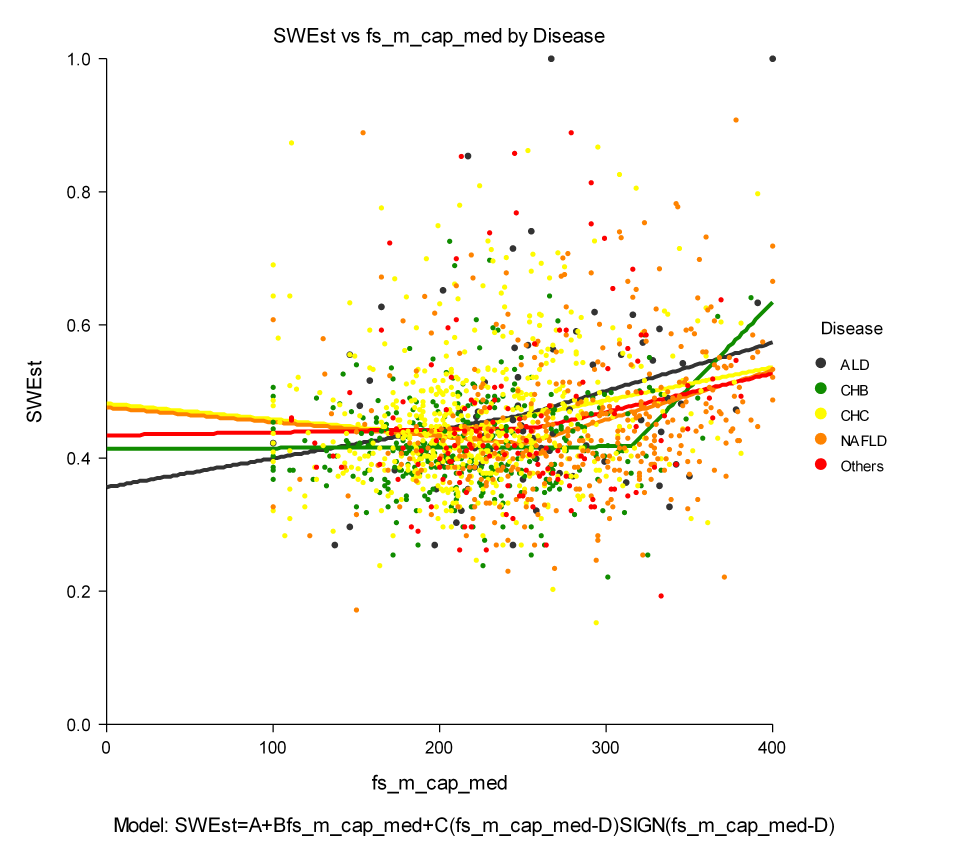

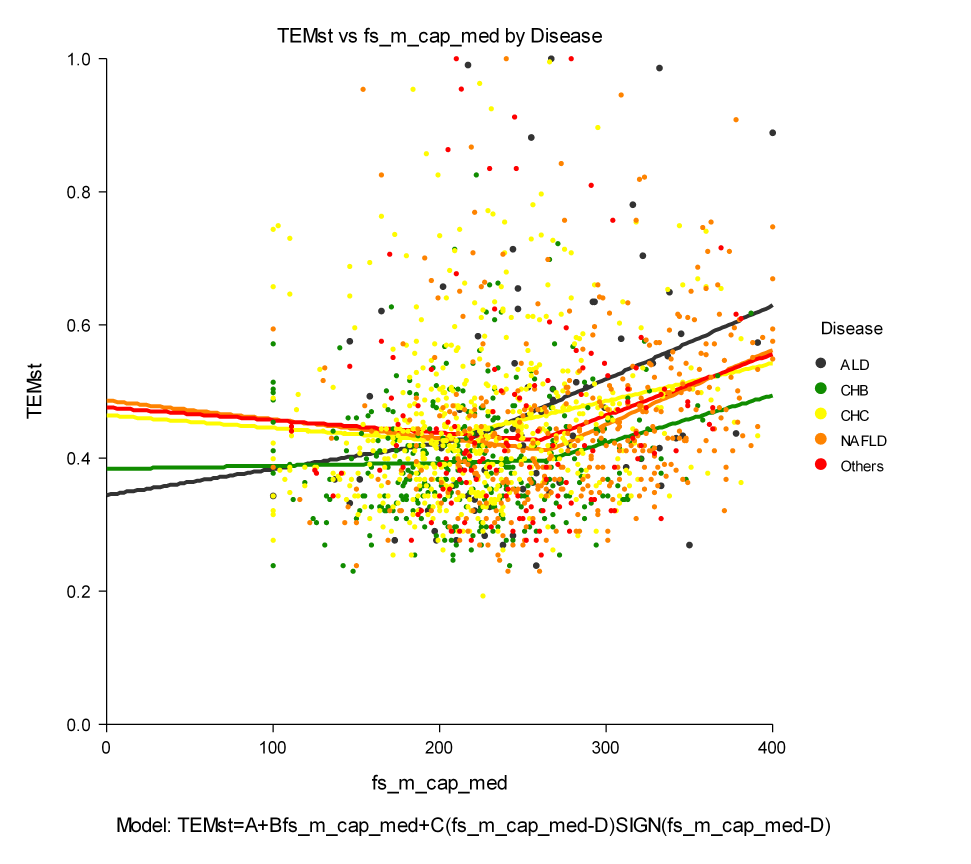

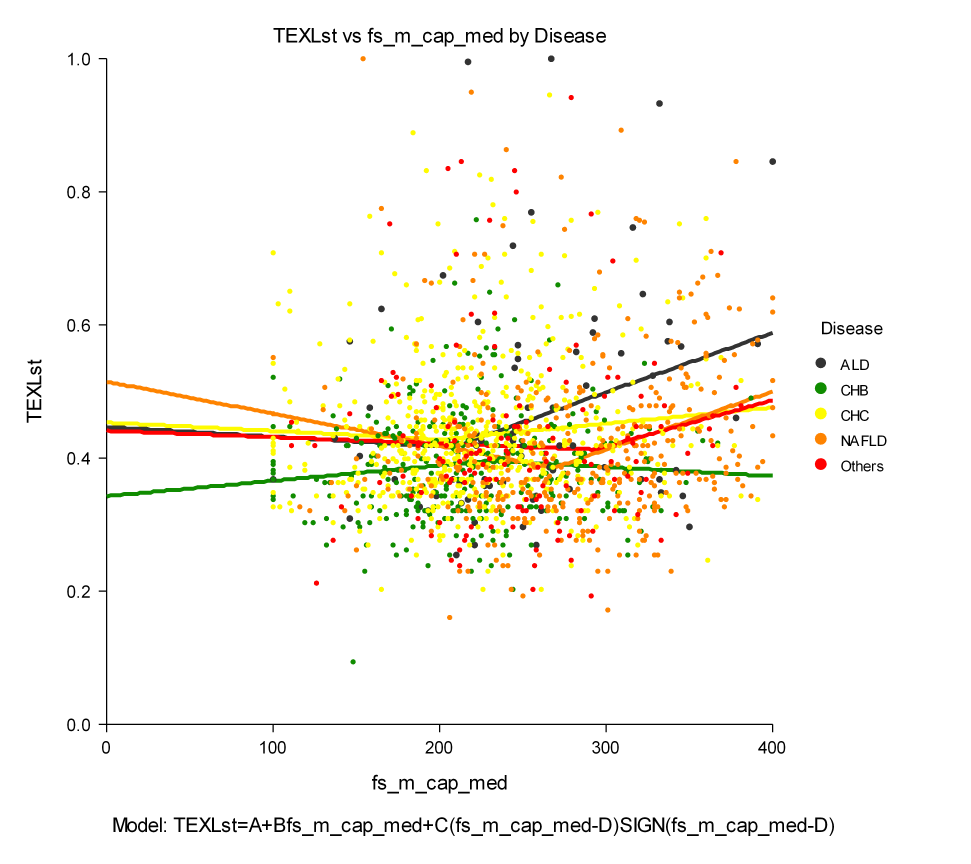

Supplement: S10 Fig — (DOCX) [file pone.0163276.s010.docx]
