# Supplementary material for: Real-Time Shear Wave versus Transient Elastography for Predicting Fibrosis: Applicability, and Impact of Inflammation and Steatosis. A Non-Invasive Comparison
Source: PLoS One. 2016 Oct 5;11(10):e0163276. doi: 10.1371/journal.pone.0163276 (PMC5051706; doi:10.1371/journal.pone.0163276)
Supplement: S1 File — (DOCX) [file pone.0163276.s011.docx]

**S1 File. Detailed methods concerning patients**

Consecutive patients undergoing chronic liver disease assessment at the "Groupe Hospitalier Pitié Salpêtrière" Hospital in Paris, France were recruited. We included patients aged 18 years or older who had undergone simultaneous serum sampling for FibroTest and attempted liver stiffness measurements with 2D-SWE and TE-M and TE-XL. Standard definitions of chronic liver disease etiologies were used for alcoholic liver disease (ALD), chronic viral hepatitis B (CHB) and C (CHC) and (NAFLD) with standard definition of metabolic syndrome. [Eckel RH, Grundy SM, Zimmet PZ. The metabolic syndrome. Lancet 2005;365:1415–1428.]

Liver fibrosis stages was presumed by the fibrosis biomarkers using fibrosis scoring system, similar to the METAVIR scoring system as previously described. [Poynard T, Munteanu M, Luckina E, et al. Liver fibrosis evaluation using real-time shear wave elastography: applicability and diagnostic performance using methods without a gold standard. J Hepatol 2013;58:928-935., Poynard T, de Ledinghen V, Zarski JP, et al. Relative performances of FibroTest, Fibroscan and biopsy for assessing the stage of liver fibrosis in patients with chronic hepatitis C: a step toward the truth in the absence of a gold standard. J Hepatol 2012;56:541-548.]

**Choice of populations according to the endpoint**

The "investigated population" included all patients in who were simultaneously prescribed the four fibrosis estimates (2D-SWE, TE-M, TE-XL and FibroTest). These patients were analyzed in different subpopulations for each specific aim. **(Figure 1)** As FibroTest was taken as the reference, patients with not-reliable or FibroTest failure were excluded and the remaining patients constituted the "intention-to-diagnose population" permitting the pragmatic assessment of performances taking into account the applicability of each test. After exclusion of patients with not-reliable or failure for TE-M, and TE-XL, and failure of 2D-SWE, the remaining population constituted the "SWE reliability population" permitting to assess the 2D-SWE reliability criteria and the appropriate cutoffs. After exclusion of the not-reliable 2D-SWE the remaining patients with applicable 2D-SWE, TE-XL, TE-M and FibroTest (no-failure and reliable) permitted the concordance analyses and the performance assessment in per-protocol including impact of inflammation using ActiTest ("concordance population"). The remaining population including the patients with steatosis estimates constituted the "concordance population with SteatoTest", permitting to assess the impact of steatosis on elasticity values and on concordance rates.
