# Supplementary material for: Real-Time Shear Wave versus Transient Elastography for Predicting Fibrosis: Applicability, and Impact of Inflammation and Steatosis. A Non-Invasive Comparison
Source: PLoS One. 2016 Oct 5;11(10):e0163276. doi: 10.1371/journal.pone.0163276 (PMC5051706; doi:10.1371/journal.pone.0163276)
Supplement: S2 File — (DOCX) [file pone.0163276.s012.docx]

**S2 File. Detailed methods concerning biomarkers**

***Elasticity measurements***

2D-SWE was performed using the Aixplorer^TM^ ultrasound system (Supersonic Imagine S.A., Aix-en-Provence, France) with a convex broadband probe (SC6-1) as recommended. Shear waves are created in liver tissue from the acoustic radiation force generated by focalized ultrasound pulses. By placing a circular ROI in a 2D-SWE image, the mean and standard deviation of the elasticity within the ROI can be displayed. For each patient the mean, and the median of Qbox elasticity were assessed, as well as the lowest and the highest elasticity values. A single estimate of the Qbox elasticity was performed, as it has been previously validated using biopsy that less than five measures were sufficient for 2D-SWE in comparison with TE, three measures and finally one measure [24]. We used an 2D-SWE box size of 3.5 x 2.5 cm and ROI between 15 to 30 mm diameter. 2D-SWE measurements were performed on the right lobe of the liver through intercostal spaces with the patient in the supine position and the right arm maximally abducted. The same intercostal space was used for both the TE and 2D-SWE measurements, with 2D-SWE successively performed after TE-M and TE-XL. The upper edge of the 2D-SWE box was placed 1.5-2 cm from Glisson’s capsule in the liver and in an area of parenchyma free of large vessels. The entire real-time 2D-SWE examinations lasted approximately 5 minutes per patient.

TE-M and TE-XL were performed using M and XL probes respectively, using FibroScan^TM^ (Echosens, Paris, France) according to the instructions and training provided by the manufacturer. Only experienced operators (more than 50 2D-SWE, TE-M and TE-XL measurements) participated in the study (TPh, AN, DE, EL, HP, LB, LF, LR, MA, MM, NS, and YN).

Steatosis was also assessed using the controlled attenuation parameter (CAP) of TE-M measures. CAP was computed only when the associated liver stiffness measurement was valid and using the same signals as the one used to measure liver stiffness, The final CAP value was the median of individual CAP values and was expressed in dB/m. [25].

***Blood tests***

FibroTest, ActiTest and SteatoTest (BioPredictive Paris, France; FibroSURE LabCorp Burlington, NC, USA) were algorithms including the serum concentrations of alpha 2-macroglobulin, apolipoprotein A1, haptoglobin, total bilirubin and GGT, adjusted for age and gender. In addition to these five components, SteatoTest includes the serum concentrations of ALT, fasting glucose, triglycerides and cholesterol, adjusted for age, gender and BMI, while ActiTest, a biomarker of hepatic necrotico-inflammatory lesions, includes FibroTest components plus ALT. Alpha-2-macroglobulin, apolipoprotein A1 and haptoglobin were measured using an automatic nephelometer BNII (Dade Behring, Marburg, Germany).The recommended pre-analytical and analytical procedures were applied. The scores of these biomarkers range from 0 to 1.00, the highest scores being attributed to the most severe lesions.

***Cutoffs***

The semi-quantitative analysis used predetermined cut-offs equivalent to the standard cut-offs for METAVIR stages; for FibroTest: F0 (0 to ⩽0.28), F1 (>0.28 to ⩽0.48), F2 (>0.48 to ⩽0.58), F3 (>0.58 to ⩽0.74) and F4 (>0.74 to ⩽1). For binary comparisons we used for fibrosis the standard cutoff used for reimbursement of direct acting antiviral drugs in CHC, presumed severe fibrosis (F3) and cirrhosis (F4) (FibroTest >0.58 METAVIR F3F4).[27,28] For TE-M F0 (0 to ⩽5 kPa), F1 (>5 to ⩽7.1 kPa), F2 (>7.1 to ⩽9.5 kPa), F3 (>9.5 to ⩽12.5 kPa) and F4.1 (>12.5 to ⩽75.0 kPa).[5, 20,21,26,27,28]. For TE-XL and 2D-SWE, in the absence of consensual cutoffs,[5] We took the same cutoffs than for TE-M. For 2D-SWE, values between 75 to 300kPa were truncated at 75kPa.

For grading activity, the ActiTest cutoffs were those recommended for manufacturer since the first validation using biopsy: 0.29, 0.52 and 0.62 for A1, A2 and A3 respectively, significant activity being defined as A2A3. For grading steatosis, the SteatoTest cutoffs cutoffs were those recommended for manufacturer since the first validation using biopsy: 0.29, 0.57 and 0.69 for S1 (steatosis 1-5%), S2 (>5%-32%) and S3S4 (>32%) respectively, significant steatosis being defined as S2S3S4.
