# Supplementary material for: Real-Time Shear Wave versus Transient Elastography for Predicting Fibrosis: Applicability, and Impact of Inflammation and Steatosis. A Non-Invasive Comparison
Source: PLoS One. 2016 Oct 5;11(10):e0163276. doi: 10.1371/journal.pone.0163276 (PMC5051706; doi:10.1371/journal.pone.0163276)
Supplement: S3 File — (DOCX) [file pone.0163276.s013.docx]

**S3 File. Review of ActiTest validations**

**Omitted ActiTest validations**

ActiTest also has been extensively validated as it was measured together with FibroTest and is the only blood tests with diagnostic performance greater than transaminases for the prediction of necro-inflammatory histologic activity. Validation studies were not easily identified by the tag "ActiTest" in PubMed, due to predominance of FibroTest tag. One example of omitted evidence based was the largest validation of ActiTest in 1,459 patients of a prospective trial in CHC with biopsies, that included for the individual analysis, 1,250 patients and for the standard meta-analysis 2,017 patients, that is total of 4,726 patients evaluated with same cutoffs and standard histological scoring system.

**ActiTest publications retrieved Febuary 12th 2016 using ActiTest as key word.**

1: Gudowska M, Wojtowicz E, Cylwik B, Gruszewska E, Chrostek L. The Distribution of Liver Steatosis, Fibrosis, Steatohepatitis and Inflammation Activity inAlcoholics According to FibroMax Test. Adv Clin Exp Med. 2015 Sep-Oct;24(5):823-7. doi: 10.17219/acem/28485. PubMed PMID: 26768633.

2: Poynard T, Deckmyn O, Munteanu M, Ngo Y, Drane F, Castille JM, Housset C, Ratziu V; FIBROFRANCE Group. Awareness of the severity of liver disease re-examined using software-combined biomarkers of liver fibrosis andnecroinflammatory activity. BMJ Open. 2015 Dec 23;5(12):e010017. doi:10.1136/bmjopen-2015-010017. PubMed PMID: 26700292; PubMed Central PMCID:PMC4691773.

3: Traoré F, Gormally E, Villar S, Friesen MD, Groopman JD, Vernet G, Diallo S,Hainaut P, Maiga MY. Molecular characteristics of Hepatitis B and chronic liver disease in a cohort of HB carriers from Bamako, Mali. BMC Infect Dis. 2015 Apr 11;15:180. doi: 10.1186/s12879-015-0916-x. PubMed PMID: 25886382; PubMed Central PMCID: PMC4403772.

4: Yakoob R, Bozom IA, Thandassery RB, Rahman MO, Derbala MF, Mohannadi MJ, John AK, Sharma M, Wani H, Kaabi SA. Noninvasive biomarkers FibroTest and ActiTest versus liver biopsy in chronic hepatitis C patients: the Middle East experience. Ann Gastroenterol. 2015 Apr-Jun;28(2):265-270. PubMed PMID: 25830472; PubMed Central PMCID: PMC4367218.

5: Poynard T, Vergniol J, Ngo Y, Foucher J, Thibault V, Munteanu M, Merrouche W, Lebray P, Rudler M, Deckmyn O, Perazzo H, Thabut D, Ratziu V, de Ledinghen V; FibroFrance Study Group and the Bordeaux HBV Study Group. Staging chronic hepatitis B into seven categories, defining inactive carriers and assessing treatment impact using a fibrosis biomarker (FibroTest®) and elastography (FibroScan®). J Hepatol. 2014 Nov;61(5):994-1003. doi: 10.1016/j.jhep.2014.06.027. Epub 2014 Jul 10. PubMed PMID: 25016224.

6: Awad Mel-D, Shiha GE, Sallam FA, Mohamed A, El Tawab A. Evaluation of liver stiffness measurement by fibroscan as compared to liver biopsy for assessment of hepatic fibrosis in children with chronic hepatitis C. J Egypt Soc Parasitol. 2013 Dec;43(3):805-19. PubMed PMID: 24640880.

7: White DL, Tavakoli-Tabasi S, Kanwal F, Ramsey DJ, Hashmi A, Kuzniarek J, Patel P, Francis J, El-Serag HB. The association between serological and dietaryvitamin D levels and hepatitis C-related liver disease risk differs in African American and white males. Aliment Pharmacol Ther. 2013 Jul;38(1):28-37. doi:10.1111/apt.12341. Epub 2013 May 26. PubMed PMID: 23710689; PubMed Central PMCID:PMC3742078.

8: de Lédinghen V, Vergniol J, Barthe C, Foucher J, Chermak F, Le Bail B, Merrouche W, Bernard PH. Non-invasive tests for fibrosis and liver stiffness predict 5-year survival of patients chronically infected with hepatitis B virus. Aliment Pharmacol Ther. 2013 May;37(10):979-88. doi: 10.1111/apt.12307. Epub 2013Apr 5. PubMed PMID: 23557139.

9: Tyson GL, Richardson PA, White DL, Kuzniarek J, Ramsey DJ, Tavakoli-Tabasi S, El-Serag HB. Dietary fructose intake and severity of liver disease in hepatitis Cvirus-infected patients. J Clin Gastroenterol. 2013 Jul;47(6):545-52. doi:10.1097/MCG.0b013e31827244d9. PubMed PMID: 23426443; PubMed Central PMCID:PMC3664139.

10: Poynard T, Bruix J, Schiff ER, Diago M, Berg T, Moreno-Otero R, Lyra AC, Carrilho F, Griffel LH, Boparai N, Jiang R, Burroughs M, Brass CA, Albrecht JK. Improved inflammatory activity with peginterferon alfa-2b maintenance therapy in non-cirrhotic prior non-responders: a randomized study. J Hepatol. 2013 Mar;58(3):452-9. doi: 10.1016/j.jhep.2012.11.001. Epub 2012 Nov 14. PubMed PMID: 23159770.

11: White DL, Tavakoli-Tabasi S, Kuzniarek J, Ramsey DJ, El-Serag HB. Racial differences in the association between adiposity measures and the risk of hepatitis C-related liver disease. J Clin Gastroenterol. 2012 Oct;46(9):779-88. PubMed PMID: 22955261; PubMed Central PMCID: PMC3437036.

12: Chen SH, Li YF, Lai HC, Kao JT, Peng CY, Chuang PH, Su WP, Chiang IP. Effects of patient factors on noninvasive liver stiffness measurement using acoustic radiation force impulse elastography in patients with chronic hepatitis C. BMC Gastroenterol. 2012 Aug 10;12:105. doi: 10.1186/1471-230X-12-105. PubMed PMID: 22877310; PubMed Central PMCID: PMC3462151.

13: White DL, Hashmi A, Ramsey DJ, Kuzniarek J, Tavakoli-Tabasi S, El-Serag HB. inasteride and methadone use and risk of advanced hepatitis C related liver disease. Dig Dis Sci. 2012 Nov;57(11):3004-10. doi: 10.1007/s10620-012-2231-3.Epub 2012 Jun 5. PubMed PMID: 22669204.

14: Poynard T, Lassailly G, Diaz E, Clement K, Caïazzo R, Tordjman J, Munteanu M, Perazzo H, Demol B, Callafe R, Pattou F, Charlotte F, Bedossa P, Mathurin P, Ratziu V; FLIP consortium. Performance of biomarkers FibroTest, ActiTest, SteatoTest, and NashTest in patients with severe obesity: meta analysis of individual patient data. PLoS One. 2012;7(3):e30325. doi: 10.1371/journal.pone.0030325. Epub 2012 Mar 14. PubMed PMID: 22431959; PubMed Central PMCID: PMC3303768.

15: Usluer G, Erben N, Aykin N, Dagli O, Aydogdu O, Barut S, Cevik F, Ormen B; Study Group. Comparison of non-invasive fibrosis markers and classical liver biopsy in chronic hepatitis C. Eur J Clin Microbiol Infect Dis. 2012 Aug;31(8):1873-8. doi: 10.1007/s10096-011-1513-6. Epub 2012 Jan 11. PubMed PMID: 22231498.

16: White DL, Tavakoli-Tabasi S, Kuzniarek J, Pascua R, Ramsey DJ, El-Serag HB. Higher serum testosterone is associated with increased risk of advanced hepatitis C-related liver disease in males. Hepatology. 2012 Mar;55(3):759-68. doi: 10.1002/hep.24618. Epub 2011 Dec 14. PubMed PMID: 21858849; PubMed Central PMCID: PMC3399504.

17: Lassailly G, Caiazzo R, Hollebecque A, Buob D, Leteurtre E, Arnalsteen L, Louvet A, Pigeyre M, Raverdy V, Verkindt H, Six MF, Eberle C, Patrice A, Dharancy S, Romon M, Pattou F, Mathurin P. Validation of noninvasive biomarkers (FibroTest, SteatoTest, and NashTest) for prediction of liver injury in patients with morbid obesity. Eur J Gastroenterol Hepatol. 2011 Jun;23(6):499-506. doi: 10.1097/MEG.0b013e3283464111. PubMed PMID: 21499110.

18: Costa JM, Telehin D, Munteanu M, Kobryn T, Ngo Y, Thibault V, Joseph M, Ratziu V, Benhamou Y, Koz'ko V, Dubins'ka G, Poveda JD, Poynard T.HCV-GenoFibrotest: a combination of viral, liver and genomic (IL28b, ITPA, UGT1A1) biomarkers for predicting treatment response in patients with chronic hepatitis C. Clin Res Hepatol Gastroenterol. 2011 Mar;35(3):204-13. doi: 10.1016/j.clinre.2011.01.005. Epub 2011 Feb 26. PubMed PMID: 21354889.

19: El Guesiry D, Moez P, Hossam N, Kassem M. Usefulness of non-invasive serum markers for predicting liver fibrosis in Egyptian patients with chronic HCV infection. Egypt J Immunol. 2011;18(2):1-12. PubMed PMID: 23082465.

20: Canbakan M, Senturk H, Canbakan B, Toptas T, Tabak O, Ozaras R, Tabak F, Balcı H, Sut N, Ozbay G. Validation of biochemical markers for the prediction of liver fibrosis and necroinflammatory activity in hemodialysis patients with chronic hepatitis C. Nephron Clin Pract. 2011;117(3):c289-95. doi: 10.1159/000320751. Epub 2010 Sep 18. PubMed PMID: 20847572.

21: Moussalli J, Delaquaize H, Boubilley D, Lhomme JP, Merleau Ponty J, Sabot D, Kerever A, Valleur M, Poynard T. Factors to improve the management of hepatitis C in drug users: an observational study in an addiction centre. Gastroenterol Res Pract. 2010;2010. pii: 261472. doi: 10.1155/2010/261472. Epub 2010 Jul 18. PubMed PMID: 20811482; PubMed Central PMCID: PMC2926583.

22: Uyar C, Akcam FZ, Ciris M, Kaya O, Kockar C, Isler M. Comparison of FibroTest-ActiTest with histopathology in demonstrating fibrosis and necroinflammatory activity in chronic hepatitis B and C. Indian J Pathol Microbiol. 2010 Jul-Sep;53(3):470-5. doi: 10.4103/0377-4929.68281. PubMed PMID: 20699505.

23: Poynard T, Munteanu M, Ngo Y, Castera L, Halfon P, Ratziu V, Imbert-Bismut F, Thabut D, Bourliere M, Cacoub P, Messous D, de Ledinghen V. ActiTest accuracy for the assessment of histological activity grades in patients with chronic hepatitis C, an overview using Obuchowski measure. Gastroenterol Clin Biol. 2010 Aug-Sep;34(6-7):388-96. doi: 10.1016/j.gcb.2010.05.001. Epub 2010 Jul 2. PubMed PMID: 20580175.

24: Sökücü S, Gökçe S, Güllüoğlu M, Aydoğan A, Celtik C, Durmaz O. The role of the non-invasive serum marker FibroTest-ActiTest in the prediction of histological stage of fibrosis and activity in children with naïve chronic hepatitis B infection. Scand J Infect Dis. 2010 Sep;42(9):699-703. doi: 10.3109/00365541003774616. PubMed PMID: 20429710.

25: El-Shabrawi MH, Mohsen NA, Sherif MM, El-Karaksy HM, Abou-Yosef H, El-Sayed HM, Riad H, Bahaa N, Isa M, El-Hennawy A. Noninvasive assessment of hepatic fibrosis and necroinflammatory activity in Egyptian children with chronic hepatitis C virus infection using FibroTest and ActiTest. Eur J Gastroenterol Hepatol. 2010 Aug;22(8):946-51. doi: 10.1097/MEG.0b013e328336ec84. PubMed PMID: 20110820.

26: Anastasiou J, Alisa A, Virtue S, Portmann B, Murray-Lyon I, Williams R. Noninvasive markers of fibrosis and inflammation in clinical practice:prospective comparison with liver biopsy. Eur J Gastroenterol Hepatol. 2010 Apr;22(4):474-80. doi: 10.1097/MEG.0b013e328332dd0a. PubMed PMID: 19887952.

27: Gressner OA, Beer N, Jodlowski A, Gressner AM. Impact of quality control accepted inter-laboratory variations on calculated Fibrotest/Actitest scores for the non-invasive biochemical assessment of liver fibrosis. Clin Chim Acta. 2009 Nov;409(1-2):90-5. doi: 10.1016/j.cca.2009.09.005. Epub 2009 Sep 10. PubMed PMID:19748500.

28: Hermeziu B, Messous D, Fabre M, Munteanu M, Baussan C, Bernard O, Poynard T, Jacquemin E. Evaluation of FibroTest-ActiTest in children with chronic hepatitis C virus infection. Gastroenterol Clin Biol. 2010 Jan;34(1):16-22. doi: 10.1016/j.gcb.2009.06.007. Epub 2009 Sep 1. PubMed PMID: 19726147.

29: Rubio A, Monpoux F, Huguon E, Truchi R, Triolo V, Rosenthal-Allieri MA, Deville A, Rosenthal E, Boutté P, Tran A. Noninvasive procedures to evaluate liver involvement in HIV-1 vertically infected children. J Pediatr Gastroenterol Nutr. 2009 Nov;49(5):599-606. doi: 10.1097/MPG.0b013e3181a15b72. PubMed PMID:19668009.

30: Smith JO, Sterling RK. Systematic review: non-invasive methods of fibrosis analysis in chronic hepatitis C. Aliment Pharmacol Ther. 2009 Sep 15;30(6):557-76. doi: 10.1111/j.1365-2036.2009.04062.x. Epub 2009 Jun 10. Review.PubMed PMID: 19519733.

31: Poynard T, Ngo Y, Marcellin P, Hadziyannis S, Ratziu V, Benhamou Y; Adefovir Dipivoxil 437 and 438 Study Groups. Impact of adefovir dipivoxil on liver fibrosis and activity assessed with biochemical markers (FibroTest-ActiTest) in patients infected by hepatitis B virus. J Viral Hepat. 2009 Mar;16(3):203-13.doi: 10.1111/j.1365-2893.2008.01065.x. Epub 2008 Oct 22. PubMed PMID: 19175871.

32: Pais R, Lupşor M, Poantă L, Silaghi A, Rusu ML, Badea R, Dumitraşcu DL. Liver biopsy versus noninvasive methods--fibroscan and fibrotest in the diagnosis of non-alcoholic fatty liver disease: a review of the literature. Rom J Intern Med. 2009;47(4):331-40. Review. PubMed PMID: 21179914.

33: Gui HL, Xie Q, Wang H. [FibroTest-ActiTest for predicting liver fibrosis and inflammatory activity in Chinese patients with chronic hepatitis B]. Zhonghua Gan Zang Bing Za Zhi. 2008 Dec;16(12):897-901. Chinese. PubMed PMID: 19105932.

34: Halfon P, Munteanu M, Poynard T. FibroTest-ActiTest as a non-invasive marker of liver fibrosis. Gastroenterol Clin Biol. 2008 Sep;32(6 Suppl 1):22-39. doi:10.1016/S0399-8320(08)73991-5. PubMed PMID: 18973844.

35: Fontanges T, Bailly F, Trepo E, Chevallier M, Maynard-Muet M, Nalet B, Beorchia S, Pillon D, Moindrot H, Froissart B, Slaoui M, Tinel X, Pradat P, Trepo C. Discordance between biochemical markers of liver activity and fibrosis (Actitest-Fibrotest) and liver biopsy in patients with chronic hepatitis C. Gastroenterol Clin Biol. 2008 Oct;32(10):858-65. doi: 10.1016/j.gcb.2008.05.019. Epub 2008 Sep 4. PubMed PMID: 18775614.

36: Zois CD, Baltayiannis GH, Karayiannis P, Tsianos EV. Systematic review: hepatic fibrosis - regression with therapy. Aliment Pharmacol Ther. 2008 Nov 15;28(10):1175-87. doi: 10.1111/j.1365-2036.2008.03840.x. Epub 2008 Aug 30. Review. PubMed PMID: 18761707.

37: Ngo Y, Benhamou Y, Thibault V, Ingiliz P, Munteanu M, Lebray P, Thabut D, Morra R, Messous D, Charlotte F, Imbert-Bismut F, Bonnefont-Rousselot D, Moussalli J, Ratziu V, Poynard T. An accurate definition of the status ofinactive hepatitis B virus carrier by a combination of biomarkers

(FibroTest-ActiTest) and viral load. PLoS One. 2008 Jul 2;3(7):e2573. doi:10.1371/journal.pone.0002573.

38: Rosenthal-Allieri MA, Tran A, Halfon P, Imbert-Bismut F, Munteanu M, Messous D, Peritore ML, Poynard T, Bernard A. Optimal correlation between different instruments for Fibrotest-Actitest protein measurement in patients with chronic hepatitis C. Gastroenterol Clin Biol. 2007 Oct;31(10):815-21. PubMed PMID: 18166859.

39: Morali G, Maor Y, Klar R, Braun M, Ben Ari Z, Bujanover Y, Zuckerman E, BogerS, Halfon P. Fibrotest-Actitest: the biochemical marker of liver fibrosis--the Israeli experience. Isr Med Assoc J. 2007 Aug;9(8):588-91. PubMed PMID: 17877064.

40: Thuluvath PJ, Krok KL. Noninvasive markers of fibrosis for longitudinal assessment of fibrosis in chronic liver disease: are they ready for prime time? Am J Gastroenterol. 2006 Jul;101(7):1497-9. Erratum in: Am J Gastroenterol. 2006 Sep;101(9):2171. Am J Gastroenterol. 2006 Oct;101(10):2445. PubMed PMID: 16863552.

41: Thabut D, Le Calvez S, Thibault V, Massard J, Munteanu M, Di Martino V, Ratziu V, Poynard T. Hepatitis C in 6,865 patients 65 yr or older: a severe and neglected curable disease? Am J Gastroenterol. 2006 Jun;101(6):1260-7. PubMed PMID: 16771947.

42: Sène D, Limal N, Messous D, Ghillani-Dalbin P, Charlotte F, Thiollière JM, Piette JC, Imbert-Bismut F, Halfon P, Poynard T, Cacoub P. Biological markers of liver fibrosis and activity as non-invasive alternatives to liver biopsy in patients with chronic hepatitis C and associated mixed cryoglobulinemia vasculitis. Clin Biochem. 2006 Jul;39(7):715-21. PubMed PMID: 16765932.

43: Férard G, Piton A, Messous D, Imbert-Bismut F, Frairi A, Poynard T, Lessinger JM. Intermethod calibration of alanine aminotransferase (ALT) and gamma-glutamyltransferase (GGT) results: application to Fibrotest and Actitest scores. Clin Chem Lab Med. 2006;44(4):400-6. PubMed PMID: 16599832.

44: Thabut D, Naveau S, Charlotte F, Massard J, Ratziu V, Imbert-Bismut F, Cazals-Hatem D, Abella A, Messous D, Beuzen F, Munteanu M, Taieb J, Moreau R, Lebrec D, Poynard T. The diagnostic value of biomarkers (AshTest) for the prediction of alcoholic steato-hepatitis in patients with chronic alcoholic liver disease. J Hepatol. 2006 Jun;44(6):1175-85. Epub 2006 Mar 13. PubMed PMID: 16580087.

45: d'Arondel C, Munteanu M, Moussalli J, Thibault V, Naveau S, Simon A, Messous D, Morra R, Blot C, Poynard T. A prospective assessment of an 'a la carte' regimen of PEG-interferon alpha2b and ribavirin combination in patients with chronic hepatitis C using biochemical markers. J Viral Hepat. 2006 Mar;13(3):182-9. PubMed PMID: 16475994.

46: Poynard T, Ratziu V, Naveau S, Thabut D, Charlotte F, Messous D, Capron D, Abella A, Massard J, Ngo Y, Munteanu M, Mercadier A, Manns M, Albrecht J. The diagnostic value of biomarkers (SteatoTest) for the prediction of liver steatosis. Comp Hepatol. 2005 Dec 23;4:10. PubMed PMID: 16375767; PubMed Central PMCID: PMC1327680.

47: Poynard T, Zoulim F, Ratziu V, Degos F, Imbert-Bismut F, Deny P, Landais P, El Hasnaoui A, Slama A, Blin P, Thibault V, Parvaz P, Munteanu M, Trepo C. Longitudinal assessment of histology surrogate markers (FibroTest-ActiTest) during lamivudine therapy in patients with chronic hepatitis B infection. Am J Gastroenterol. 2005 Sep;100(9):1970-80. PubMed PMID: 16128941.

48: Piton A, Messous D, Imbert-Bismut F, Bergès J, Munteanu M, Poynard T, Hainque B. [Alpha 2 macroglobulin immunoturbidimetric assays (DakoCytomation reagents) on Roche Diagnostic analysers (Modular P, Cobas Integra). Application to FibroTest-Actic-Test]. Ann Biol Clin (Paris). 2005 Jul-Aug;63(4):385-95. French. PubMed PMID: 16061437.

49: Imbert-Bismut F, Messous D, Raoult A, Poynard T, Bertrand JJ, Marie PA, Louis V, Audy C, Thouy JM, Hainque B, Piton A. [Results transferability on RXL, ARX, X-Pand, BN2 (Dade Behring) and modular DP (Roche Diagnostics) analysers: application to component assays of fibrotest and Actitest]. Ann Biol Clin (Paris). 2005 May-Jun;63(3):305-13. French. PubMed PMID: 15951262.

50: Poynard T, Imbert-Bismut F, Munteanu M, Messous D, Myers RP, Thabut D, Ratziu V, Mercadier A, Benhamou Y, Hainque B. Overview of the diagnostic value of biochemical markers of liver fibrosis (FibroTest, HCV FibroSure) and necrosis (ActiTest) in patients with chronic hepatitis C. Comp Hepatol. 2004 Sep 23;3(1):8. PubMed PMID: 15387887; PubMed Central PMCID: PMC522750.

51: Munteanu M, Messous D, Thabut D, Imbert-Bismut F, Jouys M, Massard J, Piton A, Bonyhay L, Ratziu V, Hainque B, Poynard T. Intra-individual fasting versus postprandial variation of biochemical markers of liver fibrosis (FibroTest) and activity (ActiTest). Comp Hepatol. 2004 Jun 23;3(1):3. PubMed PMID: 15214966; PubMed Central PMCID: PMC449730.

Since the February 2016 search, a new reference in NAFLD was published (600 patients)

Munteanu M, Tiniakos D, Anstee Q, Charlotte F, Marchesini G, Bugianesi E, et al. (2016) Diagnostic performance of FibroTest, SteatoTest, and ActiTest in patients with NAFLD using the SAF-score as histological reference. Alim Pharmacol Ther 44:877-89.
