# Supplementary material for: Real-Time Shear Wave versus Transient Elastography for Predicting Fibrosis: Applicability, and Impact of Inflammation and Steatosis. A Non-Invasive Comparison
Source: PLoS One. 2016 Oct 5;11(10):e0163276. doi: 10.1371/journal.pone.0163276 (PMC5051706; doi:10.1371/journal.pone.0163276)
Supplement: S4 File — (DOCX) [file pone.0163276.s014.docx]

**S4 File. Review of SteatoTest validations articles**

**Omitted SteatoTest validation**

We acknowledge that SteatoTest had much less studies available than FibroTest and ActiTest. Validation studies were not easily identified by the tag "ActiTest" in PubMed (Supplementary-file-S11), due to predominance of FibroTest tag. One example of omitted evidence based was the largest validation of SteatoTest in 1,415 patients of a prospective trial in CHC with biopsies [41]. Adding these patients with those of the original validation including 744 patients with different liver diseases, and 494 patients with obese patients led to 2,653 patients evaluated with same cutoffs and standard histological scoring system.

Since the February 2016 search, a new reference in NAFLD was published (600 patients)

[26]. Munteanu M, Tiniakos D, Anstee Q et al. (2016) Diagnostic performance of FibroTest, SteatoTest, and ActiTest in patients with NAFLD using the SAF-score as histological reference. Alim Pharmacol Ther, in press

**SteatoTest publications retrieved February 12th 2016 using SteatoTest as key word.**

2: Gudowska M, Wojtowicz E, Cylwik B, Gruszewska E, Chrostek L. The Distribution of Liver Steatosis, Fibrosis, Steatohepatitis and Inflammation Activity in Alcoholics According to FibroMax Test. Adv Clin Exp Med. 2015 Sep-Oct;24(5):823-7. doi: 10.17219/acem/28485. PubMed PMID: 26768633.

3: Jullian-Desayes I, Tamisier R, Zarski JP, Aron-Wisnewsky J, Launois-Rollinat SH, Trocme C, Levy P, Joyeux-Faure M, Pepin JL. Impact of effective versus sham continuous positive airway pressure on liver injury in obstructive sleep apnoea: Data from randomized trials. Respirology. 2016 Feb;21(2):378-85. doi:10.1111/resp.12672. Epub 2015 Nov 16. PubMed PMID: 26567858.

4: Copaci I, Lupescu I, Caceaune E, Chiriac G, Ismail G. Noninvasive Markers of Improvement of Liver Steatosis Achieved by Weight Reduction in Patients with Nonalcoholic Fatty Liver Disease. Rom J Intern Med. 2015 Jan-Mar;53(1):54-62. PubMed PMID: 26076562.

5: Pais R, Rusu E, Zilisteanu D, Circiumaru A, Micu L, Voiculescu M, Poynard T, Ratziu V. Prevalence of steatosis and insulin resistance in patients with chronic hepatitis B compared with chronic hepatitis C and non-alcoholic fatty liver disease. Eur J Intern Med. 2015 Jan;26(1):30-6. doi: 10.1016/j.ejim.2014.12.001. Epub 2014 Dec 29. PubMed PMID: 25553983.

6: Zelber-Sagi S, Salomone F, Webb M, Lotan R, Yeshua H, Halpern Z, Santo E, Oren R, Shibolet O. Coffee consumption and nonalcoholic fatty liver onset: a prospective study in the general population. Transl Res. 2015 Mar;165(3):428-36. doi: 10.1016/j.trsl.2014.10.008. Epub 2014 Oct 17. PubMed PMID: 25468486.

7: Perazzo H, Munteanu M, Ngo Y, Lebray P, Seurat N, Rutka F, Couteau M, Jacqueminet S, Giral P, Monneret D, Imbert-Bismut F, Ratziu V, Hartemann-Huertier A, Housset C, Poynard T; FLIP Consortium. Prognostic value of liver fibrosis and steatosis biomarkers in type-2 diabetes and dyslipidaemia. Aliment Pharmacol Ther. 2014 Nov;40(9):1081-93. doi: 10.1111/apt.12946. Epub 2014 Sep 3. PubMed PMID: 25186086.

8: Minville C, Hilleret MN, Tamisier R, Aron-Wisnewsky J, Clement K, Trocme C, Borel JC, Lévy P, Zarski JP, Pépin JL. Nonalcoholic fatty liver disease,nocturnal hypoxia, and endothelial function in patients with sleep apnea. Chest. 2014 Mar 1;145(3):525-33. doi: 10.1378/chest.13-0938. PubMed PMID: 24264333.

9: Supronowicz Ł, Wójtowicz E, Cylwik B, Gruszewska E, Chrostek L. [The diagnostic value of non-invasive biochemical biomarkers in alcohol abuse]. Pol Merkur Lekarski. 2013 Sep;35(207):148-50. Polish. PubMed PMID: 24224451.

10: Zelber-Sagi S, Webb M, Assy N, Blendis L, Yeshua H, Leshno M, Ratziu V,Halpern Z, Oren R, Santo E. Comparison of fatty liver index with noninvasive methods for steatosis detection and quantification. World J Gastroenterol. 2013 Jan 7;19(1):57-64. doi: 10.3748/wjg.v19.i1.57. PubMed PMID: 23326163; PubMed Central PMCID: PMC3542754.

11: Grattagliano I, Ubaldi E, Napoli L, Marulli CF, Nebiacolombo C, Cottone C,

Portincasa P. Utility of noninvasive methods for the characterization of nonalcoholic liver steatosis in the family practice. The "VARES" Italian multicenter study. Ann Hepatol. 2013 Jan-Feb;12(1):70-7. PubMed PMID: 23293196.

12: Poynard T, Lassailly G, Diaz E, Clement K, Caïazzo R, Tordjman J, Munteanu M, Perazzo H, Demol B, Callafe R, Pattou F, Charlotte F, Bedossa P, Mathurin P, Ratziu V; FLIP consortium. Performance of biomarkers FibroTest, ActiTest, SteatoTest, and NashTest in patients with severe obesity: meta analysis of individual patient data. PLoS One. 2012;7(3):e30325. doi: 10.1371/journal.pone.0030325. Epub 2012 Mar 14. PubMed PMID: 22431959; PubMed Central PMCID: PMC3303768.

13: de Lédinghen V, Vergniol J, Foucher J, Merrouche W, le Bail B. Non-invasive diagnosis of liver steatosis using controlled attenuation parameter (CAP) and transient elastography. Liver Int. 2012 Jul;32(6):911-8. doi: 10.1111/j.1478-3231.2012.02820.x. PubMed PMID: 22672642.

14: Lassailly G, Caiazzo R, Hollebecque A, Buob D, Leteurtre E, Arnalsteen L, Louvet A, Pigeyre M, Raverdy V, Verkindt H, Six MF, Eberle C, Patrice A, Dharancy S, Romon M, Pattou F, Mathurin P. Validation of noninvasive biomarkers (FibroTest, SteatoTest, and NashTest) for prediction of liver injury in patients with morbid obesity. Eur J Gastroenterol Hepatol. 2011 Jun;23(6):499-506. doi:10.1097/MEG.0b013e3283464111. PubMed PMID: 21499110.

15: Rubio A, Monpoux F, Huguon E, Truchi R, Triolo V, Rosenthal-Allieri MA, Deville A, Rosenthal E, Boutté P, Tran A. Noninvasive procedures to evaluate liver involvement in HIV-1 vertically infected children. J Pediatr Gastroenterol Nutr. 2009 Nov;49(5):599-606. doi: 10.1097/MPG.0b013e3181a15b72. PubMed PMID:19668009.

16: Friedrich-Rust M, Müller C, Winckler A, Kriener S, Herrmann E, Holtmeier J, Poynard T, Vogl TJ, Zeuzem S, Hammerstingl R, Sarrazin C. Assessment of liver fibrosis and steatosis in PBC with FibroScan, MRI, MR-spectroscopy, and serum markers. J Clin Gastroenterol. 2010 Jan;44(1):58-65. doi:10.1097/MCG.0b013e3181a84b8d. PubMed PMID: 19581812.

17: Imbert-Bismut F, Naveau S, Morra R, Munteanu M, Ratziu V, Abella A, Messous D, Thabut D, Benhamou Y, Poynard T. The diagnostic value of combining carbohydrate-deficient transferrin, fibrosis, and steatosis biomarkers for the prediction of excessive alcohol consumption. Eur J Gastroenterol Hepatol. 2009 Jan;21(1):18-27. doi: 10.1097/MEG.0b013e32830a4f4c. PubMed PMID: 19011575.

18: Zelber-Sagi S, Nitzan-Kaluski D, Goldsmith R, Webb M, Zvibel I, Goldiner I, Blendis L, Halpern Z, Oren R. Role of leisure-time physical activity in nonalcoholic fatty liver disease: a population-based study. Hepatology. 2008 Dec;48(6):1791-8. doi: 10.1002/hep.22525. PubMed PMID: 18972405.

19. Ratziu V, Giral P, Munteanu M, Messous D, Mercadier A, Bernard M, Morra R, Imbert-Bismut F, Bruckert E, Poynard T. Screening for liver disease using non-invasive biomarkers (FibroTest, SteatoTest and NashTest) in patients with hyperlipidaemia. Aliment Pharmacol Ther. 2007 Jan 15;25(2):207-18.

20. Poynard T, Ratziu V, Naveau S, Thabut D, Charlotte F, Messous D, Capron D, Abella A, Massard J, Ngo Y, Munteanu M, Mercadier A, Manns M, Albrecht J. The diagnostic value of biomarkers (SteatoTest) for the prediction of liver steatosis. Comp Hepatol. 2005 Dec 23;4:10.
