# Supplementary material for: Real-Time Shear Wave versus Transient Elastography for Predicting Fibrosis: Applicability, and Impact of Inflammation and Steatosis. A Non-Invasive Comparison
Source: PLoS One. 2016 Oct 5;11(10):e0163276. doi: 10.1371/journal.pone.0163276 (PMC5051706; doi:10.1371/journal.pone.0163276)
Supplement: S5 File — (DOCX) [file pone.0163276.s015.docx]

**S5 File. Details of other limitations of the present study.**

We acknowledged that despite statistical significance, the difference in curve-fitting between TE and 2D-SWE were small and could be viewed as not clinically relevant. However, the utility was not to assess the strength of concordance, the appropriate method being the LCC between tests, but to confirm graphically that the association was weak for the zone of minimal fibrosis (F0F1 presumed by Fibrotest), rose in the intermediate stages (F1F2) and was very significant for severe fibrosis (F3F4). This was observed with the three methods of elastography, for all chronic liver disease. 2D-SWE was less impacted than TEM by inflammation or activity whatever the liver disease and especially in patients with minimal fibrosis. The clinically significant impact could be interesting for the prioritization of DAA treatments in CHC and the definition of inactive carriers of HBV.

The measurements were not blinded as the same operator performed successively TE-M, TE-XL and 2D-SWE. Since 2D-SWE was performed after TE presumably by the same operator, there was a substantial risk that the knowledge of the findings at TE might influence the measurement of 2D-SWE. However, we did not increase the number of TD-SWE measurements according to previous TEM or TEDXL results. Only one measurement for 2D-SWE. Since the color box is significantly wider than the the ROI the operator could have been induced to avoid areas where the color was suggesting values discrepant from those of TE. Due to the influence of operator effect this could artificially reduced the performances of the first elastography in comparison with the following which beneficiate of the the first results or failures.

We utilized the same cut-off values for 2D-SWE and TE-XL as of TE-M in the absence of consensual cut-offs. It was not possible to use specific cutoffs for each elastography method and for each liver disease. Waiting for large evidence based studies, taking into account not only the cause of liver diseases but also the age, the gender, the necroinflammatory activity grade and steatosis grade, it was not possible nowadays to recommend specific cutoffs for each elasticity method according to each liver disease.

A limitation of the present study was the low prevalence of patients with decompensated cirrhosis as one previously observed advantage of 2D-SWE was its higher applicability in patients with ascites than for TE-M.[5] Another limitation was the absence of comparisons with automatic assessment, such as that combining CV and temporal variability.[6]

The exclusion of the patients with interval between blood tests and elasticity measurements between one to six months greater than 30 days (3%) did not change significantly the results including the LIN coefficients for activity or steatosis (Table 3). There was missing data for SteatoTest in 318 patients. These patients were similar from the patients with non-missing SteatoTest.
