# Supplementary material for: Real-Time Shear Wave versus Transient Elastography for Predicting Fibrosis: Applicability, and Impact of Inflammation and Steatosis. A Non-Invasive Comparison
Source: PLoS One. 2016 Oct 5;11(10):e0163276. doi: 10.1371/journal.pone.0163276 (PMC5051706; doi:10.1371/journal.pone.0163276)
Supplement: S1 Table — (DOCX) [file pone.0163276.s016.docx]

**S1 Table. Published definitions of failures and reliability of elasticity values assessed by 2D-SWE**

| **Applicability criteria** | **Description or comment** | **Cutoff (validation method)** |  |
| --- | --- | --- | --- |
| **Failure** |  |  | **Reference** |
| No measurement |  |  | Elkrief 2015,[8] |
| No measurement or too low signal**^1, 2^** | Minimal elasticity value too low |  | Cassinoto 2015,[13] |
| Apnea less than 5 seconds | Minimal stability of signal often related to apnea |  | Procopet 2015,[7] |
| Too heterogeneity in elasticity values | "Homogeneous" (no definition) color in a ROI of at least 15mm diameter | 15 mm ROI (Q-Box) | Sporea 2013,[17] |
| Percentage of non-filling of elasticity map (PNFE) **^2^** | Automatic selected frame over the central zone | >60% (PNFE) | Pellot-Barakat, 2015,[6] |
| **Reliability criteria and predetermined cutoffs** |  |  |  |
| Not too low elasticity value **^2^** | Too low elasticity value. Less than 1kPa recommended by manufacturer but no validation published | Minimal elasticity value ≥ 0.2 kPa (ICC, CC, AUROC) | Poynard 2013,[5] Gerber 2015,[15] |
| Variability of elasticity value **^1^** | CV of elasticity values in the ROI | CV ≤30% (ICC, CC) with biopsy. CV ≤10% (AUROC with high portal hypertension, kappa with TE-M). | Pellot-Barakat 2015,[6] Procopet 2015,[7] Elkrief 2015,[8] Bota,[9] Samir 2015,[11] Thiele 2015,[16] |
| Percentage of non-filled pixels (PNFE) | Criteria of homogeneity in ROI | Validated in combination with TV | Pellot-Barakat 2015,[6] |
| Temporal variability stability, | Automatic selected frame over the central zone | Good quality if temporal variability less than 1 kPa | Pellot-Barakat 2015,[6] |
| Spatial variability | Elasticity value CV over the whole insonation window. Correlated with elasticity | Good quality if spatial variability less than 2 kPa. | Pellot-Barakat 2015,[6] |
| Best place for region of interest | Upper part of the right lobe (segment V). | Comparison between lobes or segments (CC and AUROC with histological fibrosis stages). | Samir 2015,[11] Huang,[12] |
| Not too deep | Depth of measurement from the probe surface or from liver capsule**^3^** | <5.6 cm from the probe surface (In healthy volunteers and phantoms, success rate and CV≤10%. In patients AUROC with high portal hypertension, kappa with TE-M). | Wang 2014,[10] Procopet 2015,[7] Huang 2014,[12] |
| Not too superficial | Depth of measurement from the liver capsule | 1 to 2 cm below the liver capsule | Wang 2014,[10] Thiele 2015,[16] |
| Supine position | 2 positions compared for estimating elasticity of segment V | Comparison between supine and left decubitus position (Success rate and CV). | Huang 2014,[12] |

**^1^** Three elasticity values except for all references except for 2 authors (n=5)[15,17]. **^2^**Articles sometimes included this item as criteria of failure, or as criteria of non-reliability **^3^**In one study the depth cutoff (≤5cm) was estimated from liver capsule and not from probe surface.[10]

PNFE: Percentage of non-filling of elasticity map. CV: Standard deviation/mean-ratio (coefficient of variation). ROI= region of interest (Q-Box). ICC= Intra class correlation coefficient. CC: Spearman or Pearson correlation coefficient. AUROC: area under the receiver operating characteristics curve

2D-SWE quality criteria could be classified as either "operator-dependent" (position of transducer between the patient's ribs, placing the insonation window, freezing a stable frame and selecting a region of interest [ROI]), or "non operator-dependent" (automatic algorithms selecting the most stable frame and ROI in a registered clip on the basis of temporal and spatial homogeneity criteria such as frame surface as large as possible, and coefficient of variation as small as possible Among these quality criteria four were rational, consensual, used by the experts, recognized by the manufacturer and with a clear definition: apnea of at lest 5 seconds, probe placed in the upper part of right lobe, patient in supine position, and elasticity measured in a ROI of at least 15mm. Three criteria were consensual but without clear definitions: "Not too low signal in the ROI", "ROI not too heterogeneous", and "not too deep or not too superficial ROI" from the probe surface. Our operators followed these recommendations.
