# Supplementary material for: Real-Time Shear Wave versus Transient Elastography for Predicting Fibrosis: Applicability, and Impact of Inflammation and Steatosis. A Non-Invasive Comparison
Source: PLoS One. 2016 Oct 5;11(10):e0163276. doi: 10.1371/journal.pone.0163276 (PMC5051706; doi:10.1371/journal.pone.0163276)
Supplement: S2 Table — (DOCX) [file pone.0163276.s017.docx]

**S2 Table. List and definitions of estimates of elasticity, recorded by 2D-SWE, for each patient.**

| **Variable** | **Definition** |
| --- | --- |
| Liver L-mm (1) -distance | Distance from probe surface (skin) to top of Qbox (1 to 2 cm below the Glisson capsule) |
| Qbox Mean diameter | Qbox (ROI) mean diameter |
| Qbox Median diameter | Qbox (ROI) median diameter |
| Qbox Mean mean | Mean of elasticity mean values in Qbox |
| Qbox Median mean | Mean of elasticity median values in Qbox |
| Qbox Mean min | Mean of all minimum elasticity values in Qbox |
| Qbox Median min | Median of all minimum elasticity values in Qbox |
| Qbox Mean max | Mean of all maximum elasticity values in Qbox |
| Qbox Median max | Median of all maximum elasticity values in Qbox |
| Qbox Mean stddev | Standard Deviation of all elasticity values in Qbox |
| Qbox Median stddev | Standard Deviation of all elasticity values in Qbox |
