# Supplementary material for: Real-Time Shear Wave versus Transient Elastography for Predicting Fibrosis: Applicability, and Impact of Inflammation and Steatosis. A Non-Invasive Comparison
Source: PLoS One. 2016 Oct 5;11(10):e0163276. doi: 10.1371/journal.pone.0163276 (PMC5051706; doi:10.1371/journal.pone.0163276)
Supplement: S3 Table — (DOCX) [file pone.0163276.s018.docx]

**S3 Table. Impact of elasticity standardization criteria on concordance analyses (Lin concordance coefficient) in "concordance population with SteatoTest (n=1270).**

| **Test compared (Range)** | **2D-SWE standardization** | | |
| --- | --- | --- | --- |
|  | **Log10 range 1-75 kPa** | **Log10 range 1-300 kPa^2^** | **No Log range 1-156 kPa^2^** |
| FibroTest (0.00-1.00) | 0.275 (0.242;0.308) | 0.222 (0.195;0.249) | 0.011 (0.009;0.013) |
| TE-M Log10 unit 1-75kPa (0.00-1.00)^1^ | 0.693 (0.664;0.719) | 0.425 (0.398;0.452) | 0.010 (0.009;0.011) |
| TE-XL Log10 unit 1-75kPa (0.00-1.00) ^1^ | 0.611 (0.568;0.643) | 0.432 (0.401;0.462) | 0.008 (0.007;0.009) |
| TE-M in kPa and no Log (1-75) | 0.009 (0.008;0.009) | 0.006 (0.006;0.007) | 0.645 (0.617;0.672) |
| TE-XL in kPa and no Log (1-75) | 0.009 (0.008;0.010) | 0.007 (0.006;0.007) | 0.635 (0.603;0.666) |

^1^ Elasticity values were expressed in Log10 and standardized being divided by the maximum range (75 kPa), in order to fluctuate between 0 to 1, permitting a normal distribution and the same range than the reference FibroTest.

**^2^** 300 is the upper limit of elasticity of TD-SWE versus 75kPa for TE-M and TE-XL. The observed maximal elasticity recorded in our patients was 156 kPa.
