# Supplementary material for: Real-Time Shear Wave versus Transient Elastography for Predicting Fibrosis: Applicability, and Impact of Inflammation and Steatosis. A Non-Invasive Comparison
Source: PLoS One. 2016 Oct 5;11(10):e0163276. doi: 10.1371/journal.pone.0163276 (PMC5051706; doi:10.1371/journal.pone.0163276)
Supplement: S4 Table — (DOCX) [file pone.0163276.s019.docx]

**S4 Table. Curves fitting report according to "Minimal elasticity values".**

**Curve Fit Report**

| **Parameter Estimates for All Groups** |
| --- |
| **SWEmin4C Count Iter's R2 A B** |
| 0 132 3 0.00109 0.35150 0.05757 |
| 1 221 5 0.15897 0.01671 0.91384 |
| 2 209 5 0.14463 -0.01278 0.95941 |
| 3 1158 4 0.17397 -0.07333 1.04108 |
| Combined 1720 3 0.13788 0.02418 0.85042 |
|  |
| **Analysis of Variance Across Groups** |
| **Model Error Sum Squares Mean Square** |
| **SWEmin4C Count Iter's R2 DF Error Error** |
| 0 132 3 0.00109 130 6.52083 0.05016 |
| 1 221 5 0.15897 219 10.99162 0.05019 |
| 2 209 5 0.14463 207 9.52279 0.04600 |
| 3 1158 4 0.17397 1156 57.98437 0.05016 |
| Combined 1720 3 0.13788 1718 87.20110 0.05076 |
| Ignored 0.15945 1712 85.01960 0.04966 |
|  |
| **Curve Inequality F-Test** **Curves F-Test** |
| **Tested DF Mean Square F Ratio Prob Level** |
| All 6 0.36358 7.3213 0.00000 |
| Error 1712 0.04966 |
|  |

Classes of minimal elasticity value: 0 <0.2 kPa, 1: 0.2-0.5 kPa, 2: 0.5-1.0 kPa, and 3 >=1 kP
