# Supplementary material for: Real-Time Shear Wave versus Transient Elastography for Predicting Fibrosis: Applicability, and Impact of Inflammation and Steatosis. A Non-Invasive Comparison
Source: PLoS One. 2016 Oct 5;11(10):e0163276. doi: 10.1371/journal.pone.0163276 (PMC5051706; doi:10.1371/journal.pone.0163276)
Supplement: S5 Table — (DOCX) [file pone.0163276.s020.docx]

**S5 Table. Results of elasticity estimates of elasticity by 2D-SWE, which were recorded for each patient**

| Item | **1720 patients** |  |  | **1588 patients** |  |  |
| --- | --- | --- | --- | --- | --- | --- |
|  | mean or median | SD | CV | mean or median | SD | CV |
| Qbox (ROI) mean diameter mm | 19.0 | 2.21 | 0.12 | 19.0 | 2.23 | 0.12 |
| Qbox (ROI) median diameter mm | 19.0 | 2.20 | 0.12 | 19.0 | 2.22 | 0.12 |
| Mean of elasticity mean values in Qbox kPa | 7.646 | 5.41 | 0.707 | 7.821 | 5.53 | 0.706 |
| Mean of standardized elasticity mean values in Qbox | 0.306 | 0.205 | 0.671 | 0.448 | 0.101 | 0.226 |
| Mean of elasticity median values in Qbox | 7.647 | 5.41 | 0.707 | 7.823 | 5.53 | 0.706 |
| Mean of all minimum elasticity values in Qbox | 2.839 | 3.02 | 1.06 | 3.07 | 3.04 | 0.99 |
| Median of all minimum elasticity values in Qbox | 2.838 | 3.02 | 1.06 | 3.07 | 3.03 | 0.99 |
| Mean of all maximum elasticity values in Qbox | 15.206 | 13.73 | 0.90 | 15.146 | 13.90 | 0.92 |
| Median of all maximum elasticity values in Qbox | 15.233 | 13.75 | 0.90 | 15.175 | 13.92 | 0.92 |
| Mean Standard Deviation of all elasticity values in Qbox | 2.354 | 2.59 | 1.10 | 2.281 | 2.583 | 1.13 |

The lowest coefficient of variation was observed for "Mean of standardized elasticity mean values in Qbox"
