# Supplementary material for: Real-Time Shear Wave versus Transient Elastography for Predicting Fibrosis: Applicability, and Impact of Inflammation and Steatosis. A Non-Invasive Comparison
Source: PLoS One. 2016 Oct 5;11(10):e0163276. doi: 10.1371/journal.pone.0163276 (PMC5051706; doi:10.1371/journal.pone.0163276)
Supplement: S6 Table — (DOCX) [file pone.0163276.s021.docx]

**S6 Table. Characteristics of patients with at least one not-applicable tests (2D-SWE, TE-M, TE-XL and FT) compared to patients with the four tests applicable (concordance population).**

|  | **Not-applicable population n=663** | **Concordance population n=1,588** | **P-value** |
| --- | --- | --- | --- |
|  | n (%) or median (95% confidence interval) | n (%) or median (95% confidence interval) |  |
| **Male gender** | 362 (54.2) | 1012 (63.7) | <0.0001 |
| **Age** | 57.7 (56.9-58.8) | 54.0 (53.1-54.7) | <0.0001 |
| **Cause disease** |  |  | 0.009 |
| CHC | 225 (33.9) | 599 (37.7) |  |
| CHB | 132 (19.9) | 366 (23.0) |  |
| NAFLD | 205 (30.9) | 404 (25.4) |  |
| ALD | 24 (3.6) | 75 (4.7) |  |
| Other | 77 (11.6) | 144 (9.1) |  |
| **BMI** | 26.8 (25.9-27.7) **^1^** | 24.8 (24.6-25.1)**^9^** | <0.0001 |
| **FibroTest** | 0.38 (0.33-0.40) | 0.36 (0.34-0.38) | 0.90 |
| **ActiTest** | 0.16 (0.15-0.18) | 0.18 (0.17-0.19) | 0.003 |
| **SteatoTest** | 0.39 (0.37-0.41) **^3^** | 0.32 (0.30-0.34) **^2^** | <0.0001 |
| **2D-SWE** | 6.2 (5.9-6.4) **^8^** | 6.4 (6.3-6.5) | 0.0005 |
| **TE-M** | 6.1 (5.8-6.3) **^4^** | 6.1 (5.9-6.2) | 0.91 |
| **TE-XL** | 5.8 (5.6-6.1) **^5^** | 5.6 (5.5-5.8) | 0.001 |
| **Cap** | 234 (231-238) **^7^** | 234 (232-245) **^6^** | 0.40 |
| **Depth (mm)** | 19.4 (18.7-20.2) | 17.7 (17.4-18.0) | <0.0001 |
| **Inter-tests interval (days)** |  |  |  |
| Median | 0 (0-0) | 0 (0-0) | 0.51 |
| Range | 0-175 | 0-175 | 0.51 |
| Mean | 3.4 (2.2-4.7) | 3.6 (2.8-4.4) | 0.89 |
| Greater than 30 days | 18 (2.7) | 53 (3.3) | 0.44 |

**^1^** Missing data in 128 subjects. **^2^** Missing data in 12 subjects. **^3^** Missing data in 140 subjects.

**^4^** Missing data in 154 subjects. **^5^** Missing data in 63 subjects. **^6^** Missing data in 39 subjects.

**^7^** Missing data in 196 subjects. **^8^**Missing data in 21 subjects; **^9^**Missing data in 439 subjects;
