# Supplementary material for: Real-Time Shear Wave versus Transient Elastography for Predicting Fibrosis: Applicability, and Impact of Inflammation and Steatosis. A Non-Invasive Comparison
Source: PLoS One. 2016 Oct 5;11(10):e0163276. doi: 10.1371/journal.pone.0163276 (PMC5051706; doi:10.1371/journal.pone.0163276)
Supplement: S7 Table — (DOCX) [file pone.0163276.s022.docx]

**S7 Table. Characteristics of patients included in the** "**concordance population without ST" (1,588-1,270= 318) compared to the "concordance population" with ST (n=1,270).**

|  | **Not included in the concordance population without ST n=318** | **Included in the concordance population with ST n=1270** | **P-value** |
| --- | --- | --- | --- |
|  | **n (%) or median (95% confidence interval)** | **n (%) or median (95% confidence interval)** |  |
| **Male gender** | 223 (70.1) | 789 (62.1) | 0.008 |
| **Age** | 52.6 (50.7-54.2) | 54.4 (53.4-55.1) | 0.10 |
| **Cause disease** |  |  | <0.0001 |
| CHC | 136 (42.8) | 463 (36.5) |  |
| CHB | 70 (22.0) | 296 (23.3) |  |
| NAFLD | 42 (13.2) | 362 (28.5) |  |
| ALD | 35 (11.0) | 40 (3.1) |  |
| Other | 35 (11.0) | 109 (8.6) |  |
| **FibroTest** | 0.43 (0.37-0.47) | 0.35 (0.33-0.37) | 0.001 |
| **ActiTest** | 0.18 (0.16-0.19) | 0.17 (0.16-0.19) | 0.78 |
| **SWE** | 6.6 (6.2-6.9) | 6.4 (6.3-6.5) | 0.09 |
| **TE-M** | 6.5 (6.1-6.8) | 6.0 (5.9-6.1) | 0.003 |
| **TE-XL** | 6.1 (5.8-6.3) | 5.6 (5.5-5.8) | 0.001 |
| **Cap** | 229 (222-236) **^1^** | 237 (232-240) **^2^** | 0.08 |

**^1^** Missing data in 9 subjects. **^2^** Missing data in 30 subjects.
