# Supplementary material for: Real-Time Shear Wave versus Transient Elastography for Predicting Fibrosis: Applicability, and Impact of Inflammation and Steatosis. A Non-Invasive Comparison
Source: PLoS One. 2016 Oct 5;11(10):e0163276. doi: 10.1371/journal.pone.0163276 (PMC5051706; doi:10.1371/journal.pone.0163276)
Supplement: S8 Table — (DOCX) [file pone.0163276.s023.docx]

**S8 Table. Characteristics of patients with TE-M not-applicable tests and 2D-SWE applicable compared to patients of the "concordance population" (n=1,588).**

|  | **Not-applicable TE-M and applicable 2D-SWE n=145** | **Concordance population n=1,588** | **P-value** |
| --- | --- | --- | --- |
|  | **n (%) or median (95% confidence interval)** | **n (%) or median (95% confidence interval)** |  |
| **Male gender** | 76 (54.5) | 1012 (63.7) | 0.03 |
| **Age** | 58.8 (56.4-60.3) | 54.0 (53.1-54.7) | 0.001 |
| **Cause disease** |  |  | 0.0001 |
| CHC | 45 (31.0) | 599 (37.7) |  |
| CHB | 23 (15.9) | 366 (23.0) |  |
| NAFLD | 63 (43.5) | 404 (25.4) |  |
| ALD | 3 (2.1) | 75 (4.7) |  |
| Other | 11 (7.6) | 144 (9.1) |  |
| **BMI** | 30.0 (28.7-31.1) **^1^** | 24.8 (24.6-25.1)**^3^** | <0.0001 |
| **FibroTest** | 0.38 (0.30-0.44) | 0.36 (0.34-0.38) | 0.90 |
| **ActiTest** | 0.19 (0.15-0.22) | 0.18 (0.17-0.19) | 0.88 |
| **SteatoTest** | 0.49 (0.42-0.56) **^1^** | 0.32 (0.30-0.34) **^4^** | <0.0001 |
| **2D-SWE** | 8.1 (7.2-8.9) | 6.4 (6.3-6.5) | <0.0001 |
| **TE-M** | NA | 6.1 (5.9-6.2) | NA |
| **TE-XL** | 6.5 (6.1-7.3) | 5.6 (5.5-5.8) | <0.0001 |
| **Cap** | NA | 234 (232-245) **^6^** | NA |
| **Depth (mm)** | 22.2 (20.8-23.6) **^2^** | 17.7 (17.4-18.0)**^7^** | <0.0001 |

**^1^** Missing data in 14 subjects. **^2^** Missing data in 3 subjects. **^3^** Missing data in 311 subjects.

**^4^** Missing data in 318 subjects. **^5^** Missing data in 63 subjects. **^6^** Missing data in 39 subjects.

**^7^** Missing data in 83 subjects.
