# Supplementary material for: Real-Time Shear Wave versus Transient Elastography for Predicting Fibrosis: Applicability, and Impact of Inflammation and Steatosis. A Non-Invasive Comparison
Source: PLoS One. 2016 Oct 5;11(10):e0163276. doi: 10.1371/journal.pone.0163276 (PMC5051706; doi:10.1371/journal.pone.0163276)
Supplement: S9 Table — (DOCX) [file pone.0163276.s024.docx]

**S9 Table. Characteristics of patients with TE-XL not-applicable tests and 2D-SWE applicable compared to patients of the "concordance population" (n=1,588).**

|  | **Not-applicable TE-XL and applicable 2D-SWE n=53** | **Concordance population n=1,588** | **P-value** |
| --- | --- | --- | --- |
|  | **n (%) or median (95% confidence interval)** | **n (%) or median (95% confidence interval)** |  |
| **Male gender** | 33 (62.3) | 1012 (63.7) | 0.83 |
| **Age** | 54.6 (46.9-60.9) | 54.0 (53.1-54.7) | 0.91 |
| **Cause disease** |  |  | 0.02 |
| CHC | 9 (17.0) | 599 (37.7) |  |
| CHB | 17 (32.1) | 366 (23.0) |  |
| NAFLD | 19 (35.9) | 404 (25.4) |  |
| ALD | 1 (1.9) | 75 (4.7) |  |
| Other | 7 (13.2) | 144 (9.1) |  |
| **BMI** | 24.2 (23.1-25.5) **^1^** | 24.8 (24.6-25.1)**^3^** | 0.30 |
| **FibroTest** | 0.35 (0.20-0.50) | 0.36 (0.34-0.38) | 0.61 |
| **ActiTest** | 0.16 (0.09-0.25) | 0.18 (0.17-0.19) | 0.24 |
| **SteatoTest** | 0.29 (0.18-0.39) **^1^** | 0.32 (0.30-0.34) **^3^** | 0.16 |
| **2D-SWE** | 6.4 (5.7-7.0) | 6.4 (6.3-6.5) | 0.24 |
| **TE-M** | 5.9 (4.9-7.0) **^2^** | 6.1 (5.9-6.2) | 0.33 |
| **TE-XL** | NA | 5.6 (5.5-5.8) | NA |
| **Cap** | 229 (207-246) **^2^** | 234 (232-245) **^4^** | 0.47 |
| **Depth (mm)** | 18.3 (15.8-19.5) | 17.7 (17.4-18.0) | 0.43 |

**^1^** Missing data in 14 subjects. **^2^** Missing data in 12 subjects. **^3^** Missing data in 318 subjects.

**^4^** Missing data in 39 subjects.
