# Supplementary material for: Real-Time Shear Wave versus Transient Elastography for Predicting Fibrosis: Applicability, and Impact of Inflammation and Steatosis. A Non-Invasive Comparison
Source: PLoS One. 2016 Oct 5;11(10):e0163276. doi: 10.1371/journal.pone.0163276 (PMC5051706; doi:10.1371/journal.pone.0163276)
Supplement: S10 Table — Reliability population, n = 1720. (DOCX) [file pone.0163276.s025.docx]

**S10 Table. Comparison of the SWE coefficient of variation (ratio standard deviation/stiffness mean) cutoffs, according to concordance with the 3 other reliable tests' results. Reliability population, n= 1720.**

| **Patients’ groups according to cutoffs extracted from SWE coefficient of variation (range: 0-1)** | | | | | | | | | | | | | | | | | |
| --- | --- | --- | --- | --- | --- | --- | --- | --- | --- | --- | --- | --- | --- | --- | --- | --- | --- |
| **Cutoff (kPa)** | **1->0.500** | | | **0.375-0.500** | | | | **0.250-0.375** | | | | **0.125-0.250** | | | **<0.125** | | |
| **n** | **281** |  |  | **198** |  | | **356** | | |  | **599** | | |  | **286** | |  |
|  | **LCC Mean** | **95%CI** | | **Mean** | **95%CI** | **Mean** | | | **95%CI** | | **Mean** | | **95%CI** | | **Mean** | **95%CI** | |
| **FibroTest** | 0.161 | 0.019;0.258 | | 0.166 | 0.064;0.265 | 0.308 | | | 0.243;0.371 | | 0.318 | | 0.276;0.359 | | 0.285 | 0.225;0.342 | |
| **TE-M** | 0.459 | 0.365;0.544 | | 0.600 | 0.506;0.680 | 0.755 | | | 0.709;0.795 | | 0.780 | | 0.750;0.807 | | 0.774 | 0.728;0.813 | |
| **TE-XL** | 0.418 | 0.319;0.508 | | 0.549 | 0.446;0.637 | 0.684 | | | 0.626;0.734 | | 0.711 | | 0.672;0.747 | | 0.703 | 0.649;0.759 | |

LCC: Lin Concordance Correlation coefficient. All LCC were significant.

Despite significant lower LLC above the 0.375 CV-cutoff, this was not sufficiently discriminant to identify not-reliable 2D-SWE elasticity values, comparatively to minimal elasticity value lower than 0.2 kPa (Panel 2A).

However patients selected using CV of 2D-SWE >0.375 had lower LLC between elasticity estimated by 2D-SWE, and all the other methods of measure (FibroTest, 2D-SWE and TE-M), suggesting similar confounding variables.
