# Supplementary material for: Real-Time Shear Wave versus Transient Elastography for Predicting Fibrosis: Applicability, and Impact of Inflammation and Steatosis. A Non-Invasive Comparison
Source: PLoS One. 2016 Oct 5;11(10):e0163276. doi: 10.1371/journal.pone.0163276 (PMC5051706; doi:10.1371/journal.pone.0163276)
Supplement: S11 Table — Depth (mm) measured from probe surface to the top of the region of interest. (DOCX) [file pone.0163276.s026.docx]

**S11 Table. Comparison of the SWE depth cutoffs, according to concordance with the 3 other reliable tests' results, in the "Reliability population" (n=1,720).** Depth (mm) measured from probe surface to the top of the region of interest.

|  |  | **Patients’ groups according to SWE depth cutoffs** | | | | | | | | | |
| --- | --- | --- | --- | --- | --- | --- | --- | --- | --- | --- | --- |
| **Cutoff (kg/m^2^)** | | **Missing^1^** | | **<10mm** | | **>=10 <25mm** | | **>=25 <30mm** | | **>30mm** | |
| n | | 101 | | 176 | | 904 | | 512 | | 27 | |
|  | | **LCC Mean** | **95%CI** | **Mean** | **95%CI** | **Mean** | **95%CI** | **Mean** | **95%CI** | **Mean** | **95%CI** |
| FibroTest | | 0.337 | 0.207-0.455 | 0.249 | 0.158-0.335 | 0.289 | 0.249;0.327 | 0.216 | 0.156;0.275 | 0.199 | -0.155;0.508 |
| TE-M | | 0.482 | 0.362-0.569 | 0.619 | 0.524-0.698 | 0.732 | 0.701;0.759 | 0.534 | 0.535;0.647 | 0.310 | -0.034;0.588 |
| TE-XL | | 0.406 | 0.281-0.517 | 0.499 | 0.394-0.592 | 0.680 | 0.644;0.713 | 0.560 | 0.501;0.614 | 0.262 | -0.102;0.564 |

LCC: Lin Concordance Correlation coefficient. Only LLC among patients with 2D-SWE measure depth > 30 mm were not significant.

In the other categories, 2D-SWE was significantly associated with FibroTest without difference in the strength of concordance between the depth categories; 2D-SWE strength of concordance with TE-M and with TE-XL was higher in patients with depth >=10mm and <25mm, in comparison with patients with depth < 10mm or >=25 mm but not permitting to identify reliability cutoffs.

^1^ Patients with missing data had LLC similar values than patients with 2D-SWE value measured at depth >=25 <30mm
