# Supplementary material for: Real-Time Shear Wave versus Transient Elastography for Predicting Fibrosis: Applicability, and Impact of Inflammation and Steatosis. A Non-Invasive Comparison
Source: PLoS One. 2016 Oct 5;11(10):e0163276. doi: 10.1371/journal.pone.0163276 (PMC5051706; doi:10.1371/journal.pone.0163276)
Supplement: S12 Table — (DOCX) [file pone.0163276.s027.docx]

**S12 Table. Comparison of the WHO BMI cutoffs, according to concordance of SWE with the 3 other reliable tests' results, in the "reliability population" (n=1,720).**

|  |  | **Patients’ groups according to WHO BMI cutoffs** | | | | | | | | | |
| --- | --- | --- | --- | --- | --- | --- | --- | --- | --- | --- | --- |
| **Cutoff (kg/m^2^)** | | **Missing^1^** | | **Low <18.5** | | **Normal >=18.5-<25** | | **High >=25-<30** | | **Obese >30** | |
| n | | 338 | | 42 | | 666 | | 454 | | 220 | |
|  | | **LCC Mean** | **95%CI** | **Mean** | **95%CI** | **Mean** | **95%CI** | **Mean** | **95%CI** | **Mean** | **95%CI** |
| FibroTest | | 0.248 | 0.173;0.319 | 0.263 | 0.114;0.401 | 0.320 | 0.277;0.362 | 0.224 | 0.160;0.286 | 0.250 | 0.165;0.330 |
| TE-M | | 0.722 | 0.667;0.768 | 0.552 | 0.335;0.713 | 0.756 | 0.724;0.785 | 0.632 | 0.574;0.683 | 0.446 | 0.334;0.546 |
| TE-XL | | 0.698 | 0.640;0.749 | 0.486 | 0.247;0.669 | 0.701 | 0.662;0.737 | 0.578 | 0.514;0.635 | 0.359 | 0.244;0.465 |

LCC: Lin Concordance Correlation coefficient. All LCC were significant.

2D-SWE was significantly associated with FibroTest without difference in the strength of concordance between the BMI categories.

2D-SWE strength of concordance with TE-M and with TE-XL decreased in patients with BMI over 25 kg/m^2^, and even more in patients over 30 kg/m^2^, in comparison with patients with normal weight between 18.5 and 25 kg/m^2^.

^1^ Patients without BMI data had similar LLC between fibrosis tests than the population with normal BMI.
