# Supplementary material for: Real-Time Shear Wave versus Transient Elastography for Predicting Fibrosis: Applicability, and Impact of Inflammation and Steatosis. A Non-Invasive Comparison
Source: PLoS One. 2016 Oct 5;11(10):e0163276. doi: 10.1371/journal.pone.0163276 (PMC5051706; doi:10.1371/journal.pone.0163276)
Supplement: S13 Table — (DOCX) [file pone.0163276.s028.docx]

**S13 Table. Association between elasticity values and body mass index according to elastography method, adjusted by fibrosis severity**

| **Lin concordance coefficient between TD- SWE elasticity and BMI: mean (95% CI)** | | | |
| --- | --- | --- | --- |
|  | Elastography method | | |
| **Fibrosis severity^1^** | 2D-SWE | TE-M | TE-XL |
| All patients n=1,270 | 0.074 (0.050;0.097) | 0.107 (0.080;0.135) | 0.064 (0.034;0.093) |
| Non-severe n=989 | 0.087 (0.060;0.113) | 0.134 (0.102;0.166) | 0.070 (0.036;0.104) |
| Severe fibrosis n=281 | 0.047 (0.010;0.083) | 0.066 (0.028;0.105) | 0.051 (0.010;0.093) |

**^1^** Non-severe fibrosis are stage F0-F1-F2 presumed by FibroTest; severe are stages F3-F4.

There was no significant difference between strength of concordance (LLC) of elasticity values estimated by 2D-SWE, TE-M or TE-XL, and BMI (P>0.05).
