# Supplementary material for: Real-Time Shear Wave versus Transient Elastography for Predicting Fibrosis: Applicability, and Impact of Inflammation and Steatosis. A Non-Invasive Comparison
Source: PLoS One. 2016 Oct 5;11(10):e0163276. doi: 10.1371/journal.pone.0163276 (PMC5051706; doi:10.1371/journal.pone.0163276)
Supplement: S14 Table — (DOCX) [file pone.0163276.s029.docx]

**S14 Table. Multivariate analysis of diagnostic performance of 2D-SWE elasticity for the diagnosis of F3F4 presumed by FibroTest, adjusted on inflammation, steatosis or liver disease**

| **Model** | **Variables entered** | **Regression coefficient (95%CI)** | **AUROC (95%CI)** | **P-value vs model-1** |
| --- | --- | --- | --- | --- |
| **Model-1 baseline** | 2D-SWE | 8.49 (6.99;9.99) | 0.716 (0.678;0.751) |  |
| **Model-2** | 2D-SWE | 6.81 (5.24;8.38) | 0.793 (0.759;0.822) | <0.0001 |
|  | ActiTest | 3.87 (3.19;4.56) |  |  |
| **Model-3** | 2D-SWE | 8.67 (7.09;10.3) | 0.719 (0.680;0.753) | 0.12 |
|  | SteatoTest | -0.26 (-0.92;0.40) |  |  |
| **Model-4** | 2D-SWE | 7.89 (6.21;9.57) | 0.808 (0.776;0.836) | <0.0001 |
|  | ActiTest | 4.50 (3.75;5.26) |  |  |
|  | SteatoTest | -1.88 (-2.67;-1.09) |  |  |
| **Model-5** | 2D-SWE | 7.86 (6.21;9.57) | 0.816 (0.776;0.836) | <0.0001 |
|  | ActiTest | 4.36 (3.28;4.83) |  |  |
|  | SteatoTest | -1.80 (-2.72;-0.89) |  |  |
|  | NAFLD | -0.56 (-0.97;-0.15) |  |  |
| **Model-6** | 2D-SWE | 7.73 (6.03;9.42) | 0.811 (0.780;0.839) | <0.0001 |
|  | ActiTest | 4.15 (3.36;4.93) |  |  |
|  | SteatoTest | -1.42 (-2.26;-0.58) |  |  |
|  | CHC | 0.52 (0.19;0.86) |  |  |

Model-4 (P=0.006), model-5 (P=0.02) and model-6 (P=0.004) improved model-2 AUROCs
