# Supplementary material for: Real-Time Shear Wave versus Transient Elastography for Predicting Fibrosis: Applicability, and Impact of Inflammation and Steatosis. A Non-Invasive Comparison
Source: PLoS One. 2016 Oct 5;11(10):e0163276. doi: 10.1371/journal.pone.0163276 (PMC5051706; doi:10.1371/journal.pone.0163276)
Supplement: S15 Table — (DOCX) [file pone.0163276.s030.docx]

**S15 Table. Multivariate analysis of diagnostic performance of TE-M elasticity for the diagnosis of F3F4 presumed by FibroTest, adjusted on inflammation, steatosis or liver disease**

| **Model** | **Variables entered** | **Regression coefficient (95%CI)** | **AUROC (95%CI)** | **P-value vs model-1** |
| --- | --- | --- | --- | --- |
| **Model-1** | TE-M | 8.52 (7.17;9.86) | 0.747 (0.701;0.769) |  |
| **Model-2** | TE-M | 6.59 (5.19;7.99) | 0.800 (0.767;0.828) | P<0.0001 |
|  | ActiTest | 3.47 (2.76;4.17) |  |  |
| **Model-3** | TE-M | 9.25 (7.76;10.74) | 0.745 (0.710;0.777) | 0.04 |
|  | SteatoTest | -0.94 (-1.65;-0.25) |  |  |
| **Model-4** | TE-M | 8.08 (6.52;9.65) | 0.821 (0.791;0.847) | P<0.0001 |
|  | ActiTest | 4.21 (3.43;4.98) |  |  |
|  | SteatoTest | -2.39 (-3.21;-1.56) |  |  |
| **Model-5** | TE-M | 8.06 (6.49;9.63) | 0.820 (0.790;0.847) | P<0.0001 |
|  | ActiTest | 4.05 (3.43;4.98) |  |  |
|  | SteatoTest | -1.80 (-3.21;-1.56) |  |  |
|  | NAFLD | -0.58 (-1.01;-0.16) |  |  |
| **Model-6** | TE-M | 8.00 (6.42;9.57) | 0.824 (0.794;0.849) | P<0.0001 |
|  | ActiTest | 3.85 (3.05;4.72) |  |  |
|  | SteatoTest | -1.91 (-2.79;-1.03) |  |  |
|  | CHC | 0.55 (0.21;0.89) |  |  |

Model-4 (P=0.001), model-5 (P=0.003) and model-6 (P=0.0005) improved model-2 AUROCs
