# Supplementary material for: Real-Time Shear Wave versus Transient Elastography for Predicting Fibrosis: Applicability, and Impact of Inflammation and Steatosis. A Non-Invasive Comparison
Source: PLoS One. 2016 Oct 5;11(10):e0163276. doi: 10.1371/journal.pone.0163276 (PMC5051706; doi:10.1371/journal.pone.0163276)
Supplement: S16 Table — (DOCX) [file pone.0163276.s031.docx]

**S16 Table. Multivariate analysis of diagnostic performance of TE-XL elasticity for the diagnosis of F3F4 presumed by FibroTest, adjusted on inflammation, steatosis or liver disease**

| **Model** | **Variables entered** | **Regression coefficient (95%CI)** | **AUROC (95%CI)** | **P-value vs model-1** |
| --- | --- | --- | --- | --- |
| **Model-1** | TE-XL | 9.30 (7.83;10.77) | 0.747 (0.711;0.778) |  |
| **Model-2** | TE-XL | 7.57 (6.04;9.11) | 0.804 (0.771;0.832) | <0.0001 |
|  | ActiTest | 3.61 (2.91;4.32) |  |  |
| **Model-3** | TE-XL | 9.53 (8.00;11.6) | 0.750 (0.715;0.781) | 0.08 |
|  | SteatoTest | -0.38 (-1.06;0.29) |  |  |
| **Model-4** | TE-XL | 8.40 (6.78;10.02) | 0.819 (0.788;0.846) | <0.0001 |
|  | ActiTest | 4.23 (3.47;5.01) |  |  |
|  | SteatoTest | -1.85 (-2.64;-1.07) |  |  |
| **Model-5** | TE-XL | 8.35 (6.72;9.98) | 0.820 (0.789;0.847) | <0.0001 |
|  | ActiTest | 4.10 (3.32;4.87) |  |  |
|  | SteatoTest | -1.31 (-2.20;-0.43) |  |  |
|  | NAFLD | -0.54 (-0.96;-0.11) |  |  |
| **Model-6** | TE-XL | 8.24 (6.61;9.86) | 0.824 (0.794;0.850) | <0.0001 |
|  | ActiTest | 3.92 (3.12;4.87) |  |  |
|  | SteatoTest | -1.43 (-2.27;-0.58) |  |  |
|  | CHC | 0.49 (0.15;0.83) |  |  |

Model-4 (P=0.001), model-5 (P=0.002) and model-6 (P=0.0002) improved model-2 AUROCs
