# Supplementary material for: Real-Time Shear Wave versus Transient Elastography for Predicting Fibrosis: Applicability, and Impact of Inflammation and Steatosis. A Non-Invasive Comparison
Source: PLoS One. 2016 Oct 5;11(10):e0163276. doi: 10.1371/journal.pone.0163276 (PMC5051706; doi:10.1371/journal.pone.0163276)
Supplement: S17 Table — Impact of inflammation and steatosis severities, presumed by ActiTest (n = 599) and SteatoTest (n = 477). (DOCX) [file pone.0163276.s032.docx]

**S17 Table. Performances of elastography methods in CHC patients (n=599) for the diagnosis of severe fibrosis (F3F4) presumed by FibroTest and using a standard cutoff of 9.5 kPa.** Impact of inflammation and steatosis severities, presumed by ActiTest (n=599) and SteatoTest (n=477).

| **Elastography** | **F3F4 per elasticity** | **Se** | **Sp** | **VPP** | **VPN** | **Prevalence F3F4 per Fibrotest** |
| --- | --- | --- | --- | --- | --- | --- |
| 2D-SWE | 114/599 (19.0%) | 37.7 | 91.2 | 70.2 | 91.2 | 212/599 (35.4%) |
| TE-M | 115/599 (19.2%) | 37.3 | 90.7 | 68.7 | 72.5 | 212/599 (35.4%) |
| TE-XL | 95/599 (15.9%) | 29.2 | 91.5 | 65.3 | 70.2 | 212/599 (35.4%) |
| At least one elasticity | 164/599 (27.4%) | 47.6 | 83.7 | 61.6 | 74.5 | 212/599 (35.4%) |
| All three elasticities | 63/599 (10.5%) | 24.5 | 97.2 | 82.5 | 70.1 | 212/599 (35.4%) |
| 2D-SWE |  |  |  |  |  |  |
| ActiTest A0A1 | 66/477 (13.8%) | 29.5 | 79.1 | 54.5 | 79.1 | 122/477 (25.6%) |
| ActiTest A2A3 | 48/122 (39.3%) | 48.9 | 87.5 | 91.7 | 37.8 | 90/122 (73.8%) |
| SteatoTest S0S1 | 58/415 (14.0%) | 27.1 | 92.0 | 60.3 | 73.7 | 129/415 (31.1%) |
| SteatoTest S2S3S4 | 24/48 (50.0%) | 76.9 | 81.8 | 83.3 | 75.1 | 26/48 (54.2%) |
| TE-M |  |  |  |  |  |  |
| ActiTest A0A1 | 62/477 (13.0%) | 27.0 | 91.8 | 53.2 | 78.6 | 122/477 (25.6%) |
| ActiTest A2A3 | 53/122 (43.4%) | 51.1 | 78.1 | 86.8 | 36.2 | 90/122 (73.8%) |
| SteatoTest S0S1 | 61/415 (14.7%) | 27.9 | 91.3 | 59.0 | 73.7 | 129/415 (31.1%) |
| SteatoTest S2S3S4 | 24/48 (50.0%) | 76.9 | 81.8 | 83.3 | 75.1 | 26/48 (54.2%) |
| TE-XL |  |  |  |  |  |  |
| ActiTest A0A1 | 55/477 (11.5%) | 22.1 | 92.1 | 49.1 | 77.5 | 122/477 (25.6%) |
| ActiTest A2A3 | 40/122 (32.8%) | 38.9 | 84.4 | 87.5 | 32.1 | 90/122 (73.8%) |
| SteatoTest S0S1 | 55/415 (13.3%) | 22.5 | 90.9 | 52.7 | 72.2 | 129/415 (31.1%) |
| SteatoTest S2S3S4 | 15/48 (31.3%) | 53.8 | 95.5 | 93.3 | 63.6 | 26/48 (54.2%) |
| **Adjusted by inflammation and steatosis (n=463)** |  |  |  |  |  |  |
| 2D-SWE |  |  |  |  |  |  |
| A0A1-S0S1 n=342 | 32/342 (10.8%) | 19.5 | 91.7 | 40.5 | 79.7 | 77/342 (22.5%) |
| A2A3-S0S1 n=73 | 21/73 (28.8%) | 38.5 | 95.2 | 95.2 | 38.5 | 52/73 (71.2%) |
| A0A1-S2S3S4 n=23 | 7/23 (30.4%) | 71.4 | 87.5 | 71.4 | 87.5 | 7/23 (30.4%) |
| A2A3-S2S3S4 n= 25 | 17/25 (68.0%) | 78.9 | 66.7 | 88.2 | 50.0 | 19/25 (76.0%) |
| TE-M |  |  |  |  |  |  |
| A0A1-S0S1 n=342 | 36/342 (10.5%) | 18.2 | 91.7 | 38.9 | 79.4 | 77/342 (22.5%) |
| A2A3-S0S1 n=73 | 25/73 (34.2%) | 42.3 | 85.7 | 88.0 | 37.5 | 52/73 (71.2%) |
| A0A1-S2S3S4 n=23 | 6/23 (26.1%) | 57.1 | 87.5 | 66.7 | 82.4 | 7/23 (30.4%) |
| A2A3-S2S3S4 n= 25 | 18/25 (72.0%) | 84.2 | 66.7 | 88.9 | 57.1 | 19/25 (76.0%) |
| TE-XL |  |  |  |  |  |  |
| A0A1-S0S1 n=342 | 33/342 (9.6%) | 13.0 | 91.3 | 30.3 | 78.3 | 77/342 (22.5%) |
| A2A3-S0S1 n=73 | 31/73 (42.5%) | 53.8 | 85.7 | 90.3 | 42.9 | 52/73 (71.2%) |
| A0A1-S2S3S4 n=23 | 3/23 (13.0%) | 42.9 | 100 | 100 | 80.0 | 7/23 (30.4%) |
| A2A3-S2S3S4 n= 25 | 12/25 (48.0%) | 57.9 | 83.3 | 91.7 | 38.5 | 19/25 (76.0%) |

According to strata, the prevalences of F3F4 presumed by FibroTest varied from 22.5% in patients without severe inflammation and without severe steatosis, to 76.0% in patients with severe inflammation and severe steatosis.

Using TE-M, the number of patients above 9.5kPa (F3F4 cutoff) varied according to strata, from 10.5% in patients without severe inflammation and without severe steatosis, to 72.0% in patients with severe inflammation and severe steatosis.

Using TE-M, the number of patients above 9.5kPa (F3F4 cutoff) varied according to strata, from 10.5% in patients without severe inflammation and without severe steatosis, to 72.0% in patients with severe inflammation and severe steatosis.

Using TE-XL, the number of patients above 9.5kPa (F3F4 cutoff) varied according to strata, from 9.6% in patients without severe inflammation and without severe steatosis, to 48.0% in patients with severe inflammation and severe steatosis.
