# Supplementary material for: Real-Time Shear Wave versus Transient Elastography for Predicting Fibrosis: Applicability, and Impact of Inflammation and Steatosis. A Non-Invasive Comparison
Source: PLoS One. 2016 Oct 5;11(10):e0163276. doi: 10.1371/journal.pone.0163276 (PMC5051706; doi:10.1371/journal.pone.0163276)
Supplement: S18 Table — Analyses performed in "Intention to diagnose" in 2,239 patients, and "Per protocol" in 1,588 patients with applicable elasticity data. (DOCX) [file pone.0163276.s033.docx]

**S18 Table. Comparison of diagnostic performances of SWE, TE-M, TE-XL for the diagnosis of severe fibrosis (F3-F4) presumed by FibroTest.** Analyses performed in "Intention to diagnose" in 2,239 patients, and "Per protocol" in 1,588 patients with applicable elasticity data.

| **Biomarkers** | **n** | **SWE** | **TE-M** | **TE-XL** |
| --- | --- | --- | --- | --- |
| **AUROC model-1 (no adjustment)** |  |  |  |  |
| Intention to diagnose population | 2,239 | 0.677 (0.648;0.704) | 0.633 (0.604;0.631) | 0.704 (0.677;0.729) |
| F34 vs. F0F1F2 P-value versus SWE |  | NA | 0.004 | 0.052 |
| Per protocol population | 1,588 | 0.719 (0.686;0.749) | 0.739 (0.708;0.767) | 0.747 (0.717;0.775) |
| F34 vs. F0F1F2 P-value versus SWE |  | NA | 0.09 | 0.04 |
| **AUROC model-2 (inflammation adjustment)** |  |  |  |  |
| Intention to diagnose population | 2,239 | 0.780 (0.756;0.802) | 0.764 (0.740;0.786) | 0.784 (0.761;0.806) |
| F34 vs. F0F1F2 P-value versus SWE |  | NA | 0.01 | 0.53 |
| Per protocol population | 1,588 | 0.801 (0.773;0.826) | 0.804 (0.776;829) | 0.809 (0.782;0.833) |
| F34 vs. F0F1F2 P-value versus SWE |  | NA | 0.63 | 0.29 |

NA: Not Applicable
